# Modulation of retinoid-X-receptors differentially regulates expression of apolipoprotein genes *apoc1* and *apoeb* by zebrafish microglia

**DOI:** 10.1242/bio.058990

**Published:** 2022-02-01

**Authors:** Whitney A. Thiel, Emma J. Esposito, Anna P. Findley, Zachary I. Blume, Diana M. Mitchell

**Affiliations:** Biological Sciences, University of Idaho, Moscow, ID 83844, USA

**Keywords:** LXR-RXR, PPAR-RXR, Microglia, Apolipoproteins, Zebrafish, CNS development

## Abstract

Transcriptome analyses performed in both human and zebrafish indicate strong expression of *Apoe* and *Apoc1* by microglia. *Apoe* expression by microglia is well appreciated, but *Apoc1* expression has not been well-examined. PPAR/RXR and LXR/RXR receptors appear to regulate expression of the apolipoprotein gene cluster in macrophages, but a similar role in microglia *in vivo* has not been studied. Here, we characterized microglial expression of *apoc1* in the zebrafish central nervous system (CNS) *in situ* and demonstrate that in the CNS, *apoc1* expression is unique to microglia. We then examined the effects of PPAR/RXR and LXR/RXR modulation on microglial expression of *apoc1* and *apoeb* during early CNS development using a pharmacological approach. Changes in *apoc1* and *apoeb* transcripts in response to pharmacological modulation were quantified by RT-qPCR in whole heads, and in individual microglia using hybridization chain reaction (HCR) *in situ* hybridization. We found that expression of *apoc1* and *apoeb* by microglia were differentially regulated by LXR/RXR and PPAR/RXR modulating compounds, respectively, during development. Our results also suggest RXR receptors could be involved in endogenous induction of *apoc1* expression by microglia. Collectively, our work supports the use of zebrafish to better understand regulation and function of these apolipoproteins in the CNS.

## Abstract (precis)

**Summary:** Here we investigate expression of two apolipoprotein genes by microglia in the zebrafish model during normal development, and in contexts of pharmacological manipulations that target candidate regulatory receptors.

## INTRODUCTION

Microglia are resident leukocytes in the vertebrate central nervous system (CNS), with various roles in health and disease. In the healthy state, microglia contribute to CNS function through the clearance of dead/dying cells and debris (Blume et al., 2020; Diaz-Aparicio et al., 2016; Herzog et al., 2019; Mazaheri et al., 2014; Neumann et al., 2009; Peri and Nüsslein-Volhard, 2008; Sieger et al., 2012), synaptic pruning (Bilimoria and Stevens, 2015; Milinkeviciute et al., 2019; Paolicelli et al., 2011; Schafer et al., 2012; Scott-Hewitt et al., 2020; Weinhard et al., 2018), and regulation of neuronal populations through various mechanisms (Anderson et al., 2019; Brown and Neher, 2014; Cunningham et al., 2013; Neher et al., 2011; Sierra et al., 2010; Vilalta and Brown, 2018). It is now appreciated that nearly all CNS diseases show evidence of activation and often dysregulation of microglia, but the mechanisms by which microglia contribute to disease are not well understood. For example, in mouse models of neurodegenerative disease, a transcriptional signature has been described and attributed to so-called ‘disease associated microglia’ (Deczkowska et al., 2018; Keren-Shaul et al., 2017). This transcriptional signature shows upregulation of certain genes, many with poorly understood function, with downregulation of homeostatic genes (Deczkowska et al., 2018; Holtman et al., 2015; Keren-Shaul et al., 2017). Further, it is not clear whether the function of these disease-associated genes represents dysregulated microglial function in the diseased state, or if these genes could represent a transcriptional program that is important in controlling disease conditions.

Importantly, the function of many genes expressed by microglia, many of which have been identified in disease or degenerative conditions, remains to be determined. There is a need to better understand baseline regulation and function of microglia expressed genes, in order to understand how microglia contribute to neurodegenerative disease. Along these lines, our recent transcriptome analysis of microglia isolated from regenerating zebrafish retinal tissue (Mitchell et al., 2019) found that *apoc1* was the top hit for microglia-enriched genes. Further, *apoc1* was among the top enriched genes in phagocytic microglia isolated from adult zebrafish brain analyzed by RNA-seq (Wu et al., 2020), and is also highly enriched in microglia during acute damage response in the zebrafish brain (Oosterhof et al., 2017). At the protein level, zebrafish APOC1 was differentially regulated during retinal damage and regeneration (Eastlake et al., 2017). Together, such results indicate that this gene is crucial to some aspect of microglial function, though this function remains unknown. Interestingly, human *APOC1* variants may be associated with increased Alzheimer's disease (AD) risk (Bertram et al., 2007; Drigalenko et al., 1998; Poduslo et al., 1998; Zhou et al., 2014, 2019). Such a genetic link is also apparent, and well-appreciated, for *APOE* (Cervantes et al., 2011; Jun et al., 2012; Saunders et al., 1996; Strittmatter et al., 1993; Verghese et al., 2011), which lies just upstream of *APOC1* on chromosome 19 in humans (Mak et al., 2002; Smit et al., 1988). One report suggests an anti-inflammatory function for *APOC1*, however this function may in some way be linked to certain *APOE* alleles (Cudaback et al., 2012). In contrast, other work suggests possible *APOE*-independent effects of *APOC1* (Prendecki et al., 2018; Zhou et al., 2019). Somewhat paradoxically, both over expression and knock-out of *Apoc1* in mouse models appear to result in cognitive defects (Abildayeva et al., 2008; Berbée et al., 2011).

Similar to findings in zebrafish by transcriptome analysis, relatively strong microglial expression of *APOC1* has also been described in RNA-seq analyses of human microglia (Gosselin et al., 2017). In addition, *APOC1* is one of the most highly upregulated genes in microglia isolated from brains of human AD patients (Mathys et al., 2019; Srinivasan et al., 2020) and among the upregulated genes in aged human microglia (Olah et al., 2018). In contrast, microglial expression of *Apoc1* in mouse models is comparatively much lower (Gosselin et al., 2017). This discrepancy in expression of *Apoc1* by microglia between humans and mouse models may, at least in part, explain our current lack of understanding of *Apoc1* function as it relates to baseline microglial function in the CNS. This could also be at least part of the reason that this gene is under-studied in the CNS relative to *Apoe* and suggests that alternative models could be appropriate for studying this gene. Some previous work in zebrafish has studied *apoc1* in the early embryo during epiboly (Wang et al., 2013), but expression and regulation of this gene in the animal, after microglia colonize the developing CNS, has not yet been explored.

Although the above referenced RNA-seq experiments, including our own, indicate that microglia are a prominent cell type expressing *Apoc1* in the CNS, to our knowledge this has not been demonstrated *in situ*. Though *Apoc1* mRNA has been detected in cultured astrocytes (Petit-Turcotte et al., 2001), it is not clear if or when this is the case *in vivo*, and localization of the protein appears to occur at other locations in the CNS (Abildayeva et al., 2008; Evangelou et al., 2019; Petit-Turcotte et al., 2001). In addition, although apolipoprotein expression by peripheral macrophages has been studied in terms of lipoprotein metabolism (Fuior and Gafencu, 2019), *Apoc1* has received little attention compared to other apolipoprotein genes, most notably *Apoe*. Further, the regulation of *Apoc1* expression in microglia *in vivo* has not been studied. We considered that RXR heterodimers could be important in this regard, and that modulation of these receptors could affect *Apoc1* expression by microglia *in vivo*, given that published *in vitro* studies have examined the role of LXR-RXR and PPAR-RXR receptors in the regulation of the apolipoprotein gene cluster in macrophages (Chawla et al., 2001; Dahabreh and Medh, 2012; Mak et al., 2002; Subramanian et al., 2017). Also notable, there are reports of retinoic acid (RA) regulation of apolipoprotein genes in astrocytes (Zhao et al., 2014), indirect effects of RA on *apoc1* in the zebrafish embryo (Wang et al., 2015), as well as LXR regulation in both astrocytes and macrophages (Laffitte et al., 2001b; Liang et al., 2004; Mak et al., 2002). Considering these reports and the advantages of the zebrafish model for pharmacological manipulations via immersion and *in situ* imaging, as well as transcriptome analyses indicating conserved expression of *Apoc1* by microglia in both human and zebrafish as discussed above, the zebrafish could provide an excellent model organism to probe this gene.

Here, we confirm orthology of human *APOC1* and zebrafish *apoc1*. We show that *apoc1* expression is indeed localized to microglia in the developing zebrafish CNS and in the adult zebrafish retina. We determine that the onset of *apoc1* in a subset of microglia begins by 3 days post fertilization (dpf) and by 5 dpf most microglia express high levels of this gene. To further understand the regulation of *apoc1* expression in microglia, and to compare to that of *apoeb*, we used an *in vivo* pharmacological approach with compounds that modulate RARs, RXRs, PPAR, and LXR receptors and examined their effects on microglial expression of *apoc1* and *apoeb*. The use of zebrafish for this work allowed us to selectively modulate activity of these receptors using pharmacological immersion treatments during early CNS development. We show evidence that microglial expression of these two apolipoprotein transcripts is differentially regulated by LXR versus PPAR modulators, and that RXR receptors could be involved in endogenous regulation of microglial expression of *apoc1*. In particular we show evidence that in microglia, *apoc1* is more significantly influenced by LXR-RXR agonists than *apoeb*. In contrast, expression of *apoeb* is more significantly influenced by PPAR-RXR modulation. We show that microglia express transcripts for both *nr1h3* (*lxra*, the only *lxr* gene in zebrafish) and nr1c3 (*pparg*), suggesting that microglia could directly respond to modulation of these receptors. This suggests that future therapeutic approaches could potentially extend this work towards selective targeting of *APOC1* separate from *APOE*, if such a goal is found to be appropriate, to modulate human neurodegenerative disease in the CNS. In addition, our findings further justify the use of zebrafish as a model for future studies into the regulation and function of microglia-expressed *apoc1* in the CNS.

## RESULTS

### Orthology of human APOC1 and zebrafish apoc1

**Fig. 1.**
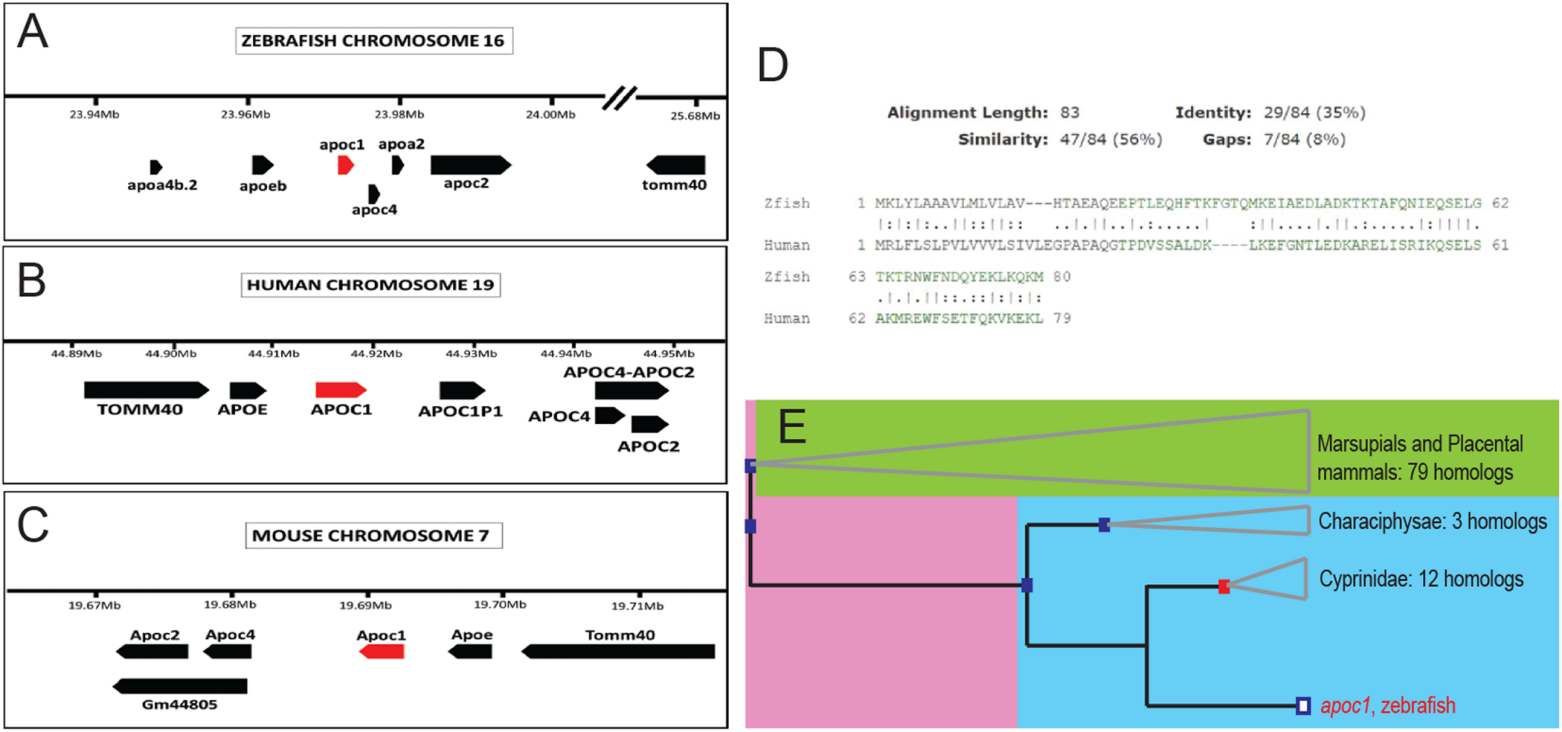
**Orthology of zebrafish *apoeb* and *apoc1* to human and mouse genes.** (A–C) Organization of the apolipoprotein gene clusters in zebrafish (A), human (B), and mouse (C). (D) Amino acid alignment of zebrafish APOC1 to human APOC1. (E) Phylogenetic relationship of zebrafish *apoc1* to the shown species as determined by ensembl.org.

We compared the chromosomal regions containing the human *APOC1* gene, mouse *Apoc1* gene, and zebrafish *apoc1* gene in the three species (Fig. 1). All three species show similar organization including chromosomal clustering of apolipoprotein genes with the *apoeb* (zebrafish; *APOE*: human, *Apoe*: mouse) gene upstream of *apoc1* in all three species (Fig. 1A–C). Other similarities include other apolipoprotein genes (*apoc2* and *apoc4*: zebrafish; *APOC2 and APOC4*: human; *Apoc2 and Apoc4*: mouse), found downstream of *apoc1* in all three species. *Tomm40* (mouse) and *TOMM40* (human) are upstream of *Apoc1* in mice and humans, but in zebrafish *tomm40* lies roughly 2Mb downstream of *apoc1*, and in the opposite orientation (Fig. 1A–C). In humans, there is also a pseudogene (*APOC1P*) downstream of *APOC1* (Lauer et al., 1988) that is not found (or not annotated) in mouse or zebrafish. Another difference in apolipoprotein gene clustering is that *apoa4b.2* is found upstream of *apoc1* in zebrafish, but in humans and mice, respectively, *APOA4* and *Apoa4* are found on a different chromosome (chromosome 11, chromosome 9).

To further examine orthologous relationship of human *APOC1* and zebrafish *apoc1*, we used the DRSC Integrative Ortholog Prediction Tool (DIOPT). DIOPT is an ortholog and paralog search tool that compares ortholog predictions from multiple algorithms, such as Compara, eggnog and OrthoDB (Hu et al., 2011). The DIOPT score was 10 for *apoc1* when comparing human and zebrafish genes (Fig. S1), indicating the human and zebrafish genes as orthologs. DIOPT analysis also showed that the orthology was ranked ‘high’ meaning that the pairs had the best scores for either forward or reverse searches and had an overall score of above 2 (Fig. S1). An amino acid alignment (UniProt) was also performed between the two species showing similarity of 56%, and conserved identity of 35% (Fig. 1D). We also used Ensembl to create a gene tree for *apoc1*, to further investigate the relationship of the zebrafish *apoc1* gene to other species. Based on this gene tree, there is a common ancestral *apoc1* gene that gave rise to both the mammalian and zebrafish genes (Fig. 1E). Collectively, we conclude that human *APOC1* and zebrafish *apoc1* are orthologs. This orthologous relationship supports that the zebrafish is an appropriate model organism to study this gene.

### *Apoc1* expression by zebrafish microglia

**Fig. 2.**
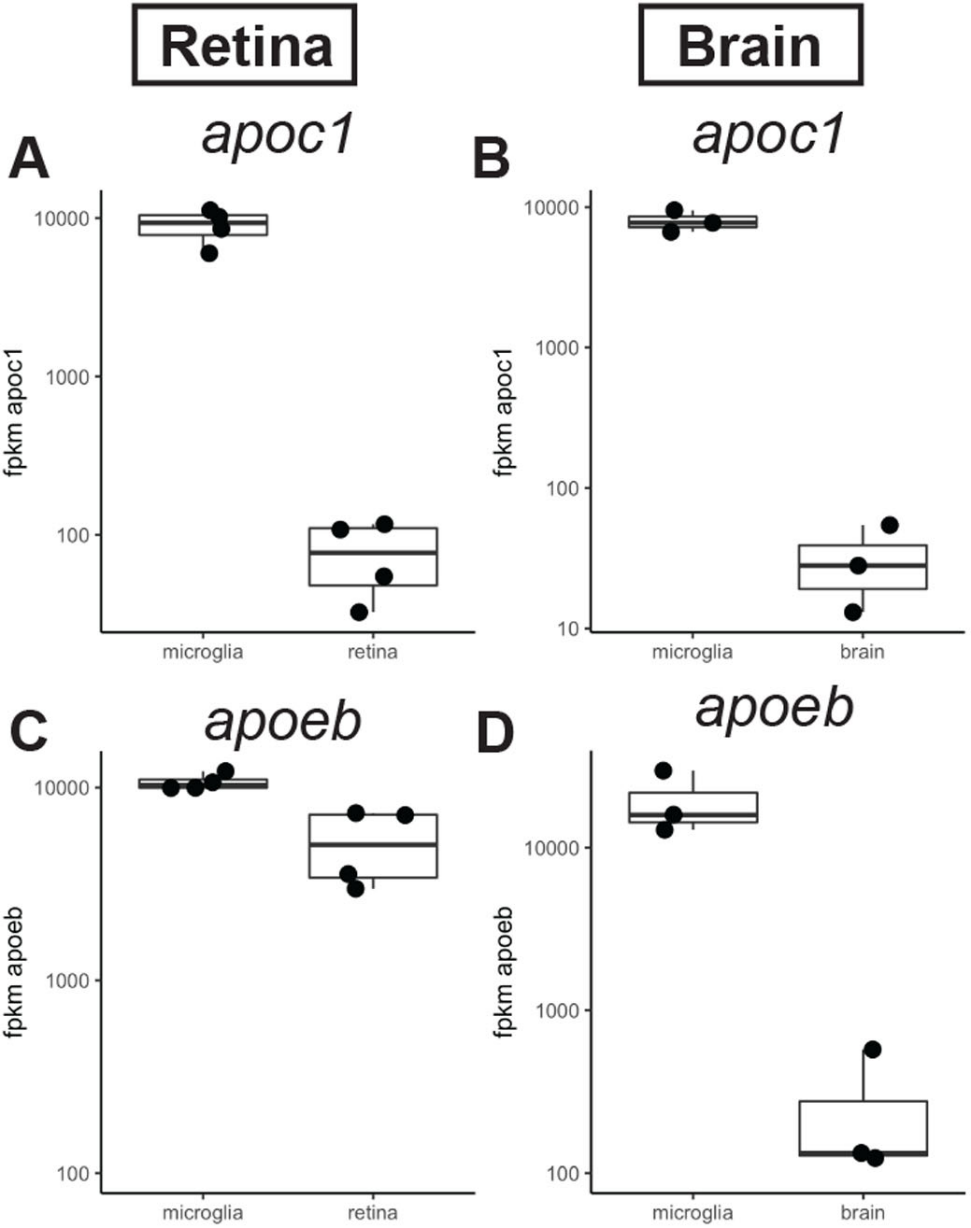
**Expression of *apoc1* and *apoeb* in the zebrafish CNS measured by RNA-seq.** (A,B) Normalized expression (fpkm, fragments per kilobase million reads) of *apoc1* in sorted populations of microglia compared other cell types isolated from regenerating zebrafish retina (A; Mitchell et al., 2019) or zebrafish brain (B; Oosterhof et al., 2017). (C,D) Normalized expression (fpkm) of *apoeb* in sorted populations of microglia compared other cell types isolated from regenerating zebrafish retina (A; Mitchell et al., 2019) or zebrafish brain (B; Oosterhof et al., 2017).

In our previous report describing the transcriptome of zebrafish microglia isolated from regenerating retinas, *apoc1* was our top hit for differentially expressed genes in microglia (Mitchell et al., 2019). We therefore used the tools at zfregeneration.org (Nieto-Arellano and Sánchez-Iranzo, 2018) to re-examine *apoc1* expression in our own work as well as another published study (Oosterhof et al., 2017), which described the transcriptome of zebrafish brain microglia. For comparison, we also examined expression of *apoeb* in this manner, a disease-associated apolipoprotein gene with well-appreciated expression by microglia in several species. In microglia isolated from regenerated retinas, *apoc1* is also highly abundant and enriched in microglia compared to other retinal cells (Fig. 2A). In the zebrafish brain, *apoc1* and *apoeb* transcripts are highly enriched in microglia when compared to other brain cells (Fig. 2B). Both microglia and other retinal cells express *apoeb* (Fig. 2C); these other *apoeb*+ retinal cells are most likely the Müller glia, for which *apoeb* expression has previously been described (Raymond et al., 2006). In the brain, *apoeb* is highly enriched in microglia with some less abundant expression in other brain cells (Fig. 2D).

In order to confirm and demonstrate unique expression of *apoc1* by microglia in the zebrafish CNS, we extracted mRNA from adult zebrafish retinas and generated cDNA by reverse transcription. To amplify cDNA corresponding to *apoc1* mRNA transcripts, we designed three primer pairs for PCR. These primer pairs hybridize in the 5′UTR/first exon and 3′UTR/last exon of a*poc1* and are expected to detect both previously described transcript variants of zebrafish *apoc1* (Wang et al., 2013). Gel electrophoresis revealed RT-PCR products at the expected size from each primer pair (Fig. S2). The cDNA amplicons were cloned and sequenced revealing identity comparisons to be 99% for all three primer pairs. We chose the product from primer pair 2 to serve as a template for generation of in-house sense and anti-sense DIG-labeled RNA probes to detect *apoc1* transcripts *in situ*. No signal was obtained from the sense probe in adult retina or embryos (Fig. S3).

We first confirmed microglial expression of *apoc1* in adult zebrafish retinas, as this was the source of mRNA for cDNA cloning. We were also interested in determining if microglia, or other cell types, express detectable *apoc1* in the undamaged adult zebrafish retina since the gene was identified in our study of retinal regeneration (Mitchell et al., 2019). Expression of *apoc1* was confirmed in adult *mpeg1*:mCherry retinas using *in situ* hybridizations followed by immunofluorescence (Fig. S4). In the adult retina, microglia express *mpeg1*-driven reporters (Mitchell et al., 2019, 2018). Nearly all *mpeg1:*mCherry*+* cells co-expressed *apoc1* (Fig. S4), though the expression of *apoc1* in each individual cell appears to be somewhat heterogenous.

**Fig. 3.**
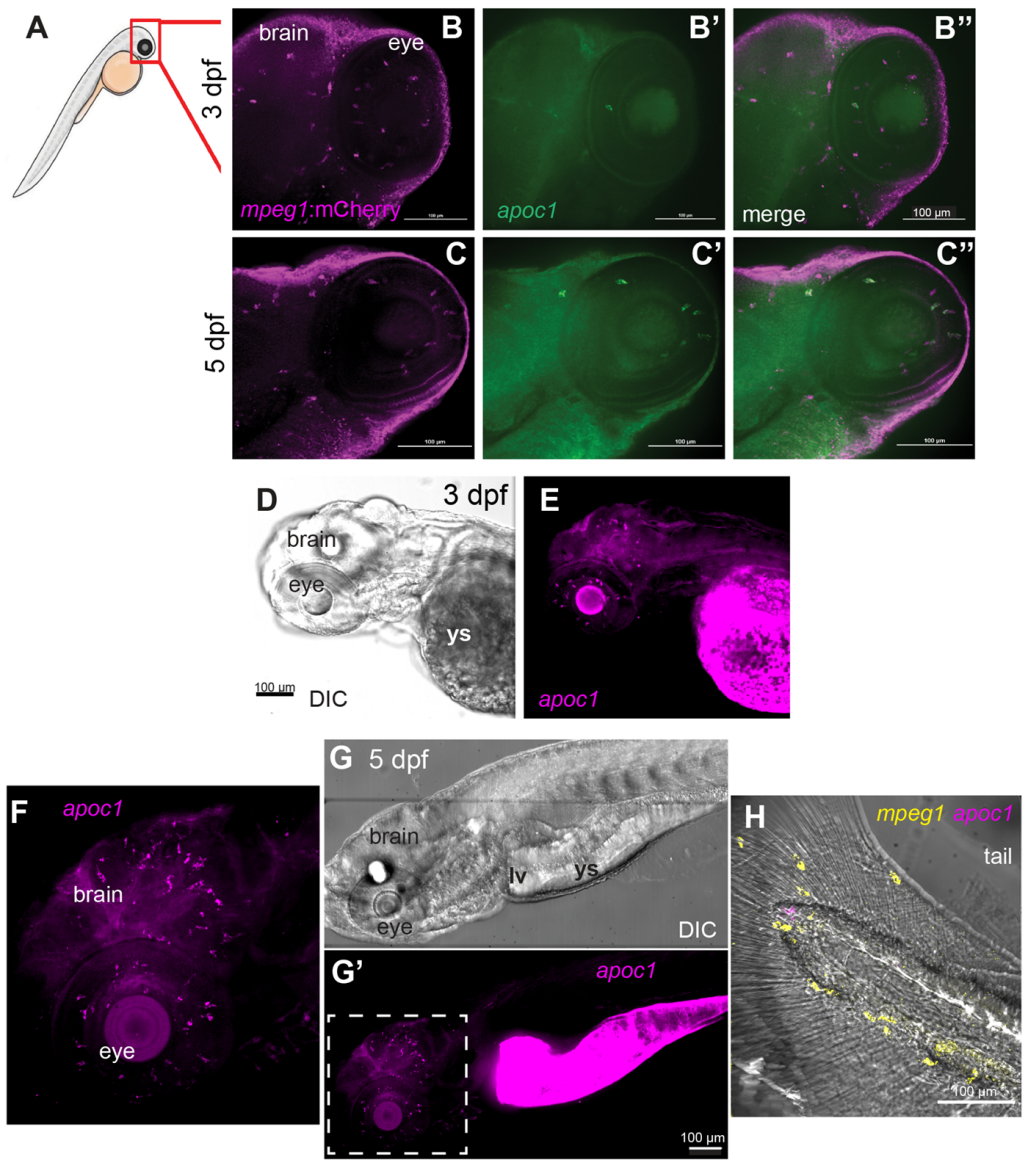
**Expression of *apoc1* in the zebrafish embryo visualized by *in situ* hybridization.** (A) Region of imaging of embryos at 3 and 5 dpf is indicated by the red box. (B–C″) *In situ* hybridization for *apoc1* using in-house generated RNA probes (green) in *mpeg1*:mCherry (magenta) transgenic embryos at the indicated ages. (D) DIC image of 3 dpf embryo. (E) Visualization of *apoc1* transcripts in the 3 dpf embryo. (F–H) Visualization of *apoc1* transcripts *in situ* in whole embryos at 5 dpf, using HCR *in situ* hybridization. (F) Fluorescent image of *apoc1* HCR probe signal showing transcripts in the head (brain and eyes). (G) DIC image of whole embryo. (G′) Fluorescent image of *apoc1* HCR probe signal showing transcripts in the head, eyes, region of remaining yolk sac (ys), and region of developing liver(lv)/gut. (F) Enlarged region indicated by dashed box in G′; transcripts in the developing CNS are consistent with microglia pattern and morphology. (H) Merged image of DIC and fluorescent HCR probe signals to detect *mpeg1* and *apoc1* in the tail. Transcripts for *apoc1* are not observed in *mpeg1*+ macrophages. Images are representative of *n*=6 embryos per timepoint.

We next examined expression of *apoc1 in situ* in embryonic zebrafish at 3 and 5 dpf, to determine if microglia express *apoc1* during early brain and retinal development. We chose these time points because microglia colonize the brain and retina by 3 dpf (Blume et al., 2020; Casano et al., 2016; Herbomel et al., 2001; Xu et al., 2016). In order to confirm that *apoc1* was expressed by microglial cells, *in situs* were first performed using *mpeg1*:mCherry fish. Co-expression of *mpeg1*:mCherry and *apoc1* confirmed that microglial cells in the developing brain and retina at both 3 and 5 dpf express *apoc1*, and *apoc1* transcripts were localized mainly to microglia (Fig. 3A–C). At 3 dpf, some *mpeg1:*mCherry+ microglia did not co-localize with *apoc1* (Fig. 3B–B″), but most microglia expressed *apoc1* by 5 dpf (Fig. 3C–C″).

We examined *apoc1* expression throughout the entire embryo, using hybridization chain reaction wholemount *in situ* hybridization (HCR WISH) (Choi et al., 2018). In 3 dpf embryos, we observed signal from the *apoc1* probe in the brain and eyes, consistent with localization of this transcript within microglia, and in the yolk sac (Fig. 3D,E) where *apoc1* expression has previously been described (Wang et al., 2013). In 5 dpf embryos, *apoc1* transcripts were also present in the region of the developing liver (Fig. 3G,G′); liver expression of *apoc1* has been described (Fuior and Gafencu, 2019). However, only brain localized macrophages (i.e. microglia) express *apoc1* (Fig. 3G′,F), as *apoc1* signal was not observed in locations of other macrophages present in the developing embryo such as the tail fin (Fig. 3H). Interestingly, this indicates that of the macrophage populations in the embryo, *apoc1* expression is restricted to microglia.

**Fig. 4.**
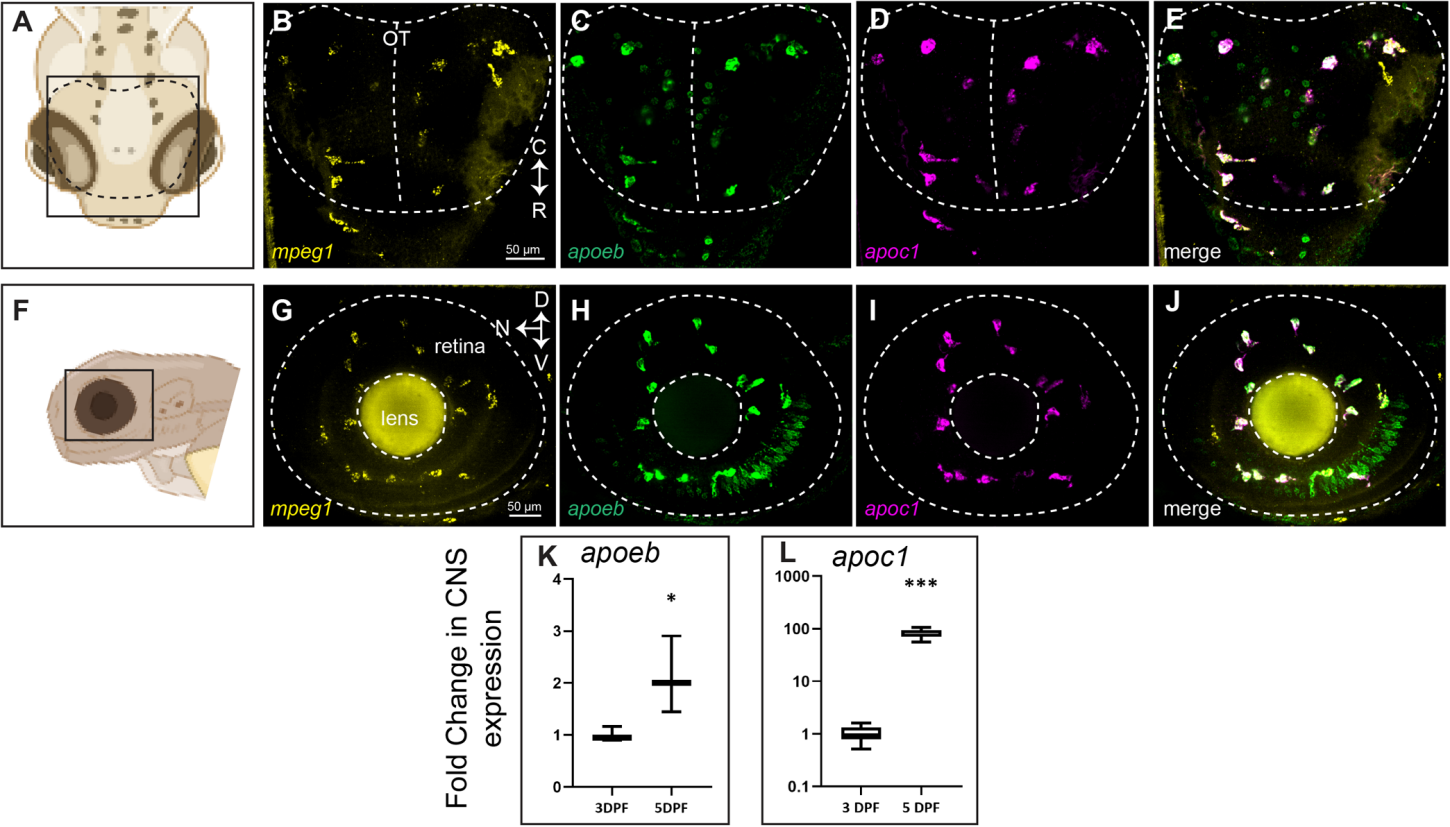
**Multiplex detection of *mpeg1*, *apoc1*, and *apoeb* transcripts in the developing zebrafish CNS.** HCR probe sets were used to detect *mpeg1*, *apoc1*, and *apoeb* transcripts in whole zebrafish embryos at 3 dpf. (A) Region and orientation of imaging of the zebrafish brain. Orientation markers: C, caudal; R, rostral. (B–D) Signal from each probe set detected within the optic tectum. (E) Merge of all three probe set signals. The region indicated by the dotted lines pertains to the optic tectum. (F) Region and orientation of imaging of the zebrafish eye/retina. Orientation markers: D, dorsal; N, nasal; V, ventral. (G–I) Signal from each probe set detected within the eye/retina. (J) Merge of all three probe set signals. The region indicated by the dotted lines pertains to the embryonic eye boundary (outer circle) as well as the lens (inner circle). In embryos, the eye is comprised nearly entirely of lens and retina. (K,L) Fold change in expression in heads from 3 to 5 dpf of *apoeb* (K) and *apoc1* (L) measured by RT-qPCR. Images in A and F were generated in BioRender. Images are representative of *n*=6 embryos.

To visualize expression of *apoc1* simultaneously with *apoeb* in the normally developing CNS, we used multiplex HCR WISH of wild-type embryos with probe sets specific for *apoc1*, *apoeb*, and *mpeg1* (Fig. 4). Both *apoc1* and *apoeb* localized with *mpeg1* in the optic tectum and retina at 3 dpf. Signal from *apoc1* was consistent with microglial morphology and almost always co-localized with *mpeg1* (Fig. 4). Consistent with *apoeb* expression both microglia and other CNS cells (Fig. 2), in addition to *mpeg1*+ cells, we also observed expression of *apoeb* in *mpeg1*- cells (Fig. 4). In the optic tectum, *apoeb*+ signal was observed in a ring-like, possibly perinuclear, pattern in cells of the optic tectum that did not co-localize with *mpeg1* (Fig. 4C,E), likely representing other glial cells or possibly neurons. Also observed and expected, *apoeb* signal was present in cells with morphological and spatial characteristics consistent with the Müller glia (Fig. 4H,J), for which *apoeb* expression has previously been described (Raymond et al., 2006). RT-qPCR revealed a nearly 100-fold increase in *apoc1* transcript levels in the heads of zebrafish between 3 and 5 dpf (Fig. 4L). This indicates that from 3 to 5 dpf, while more microglia may begin to express *apoc1*, *apoc1* transcript levels are strongly increased on a per microglial cell basis. In contrast, *apoeb* transcripts in heads increased approximately twofold (Fig. 4K) from 3 to 5 dpf.

### Effects of 9cis-RA, RXR, PPAR, and LXR modulation on apolipoprotein expression in the developing CNS

Given the strong induction of *apoc1* during early CNS development (Fig. 4K,L), we were interested in determining how microglial expression of *apoc1* may be induced, and to investigate how that may be similar or different from that of *apoeb.* Coordinate regulation of the apolipoprotein gene cluster has been reported (Cudaback et al., 2012; Evangelou et al., 2019; Mak et al., 2002). Various *in vitro* studies have examined the role of LXR-RXR and PPAR-RXR receptors in the regulation of the apolipoprotein gene cluster in macrophages and other cell types *in vitro* (Chawla et al., 2001; Dahabreh and Medh, 2012; Mak et al., 2002; Subramanian et al., 2017). Further, there are reports of RA regulation of these genes in astrocytes (Zhao et al., 2014), indirect effects of RA on *apoc1* in the zebrafish embryo (Wang et al., 2015), as well as LXR regulation in both astrocytes and macrophages (Laffitte et al., 2001b; Liang et al., 2004; Mak et al., 2002). Using in silico analysis of the ∼5 kb upstream region of zebrafish *apoc1*, we found predicted binding sites for RAR and RXR receptors, as well as a predicted PPAR-RXR site (Fig. S5). Previous work using a human astrocytoma cell line identified a PPAR response element (PPRE) in a similar region (downstream of *APOE* and upstream of *APOC1*) that was important in driving macrophage expression of *APOE* (GALETTO et al., 2001). We did not identify an LXR-RXR site in this region, though this could be because the tool performs analysis based on human sequences, or that important LXR sites are located outside of this 5 kb region (Mak et al., 2002).

**Fig. 5.**
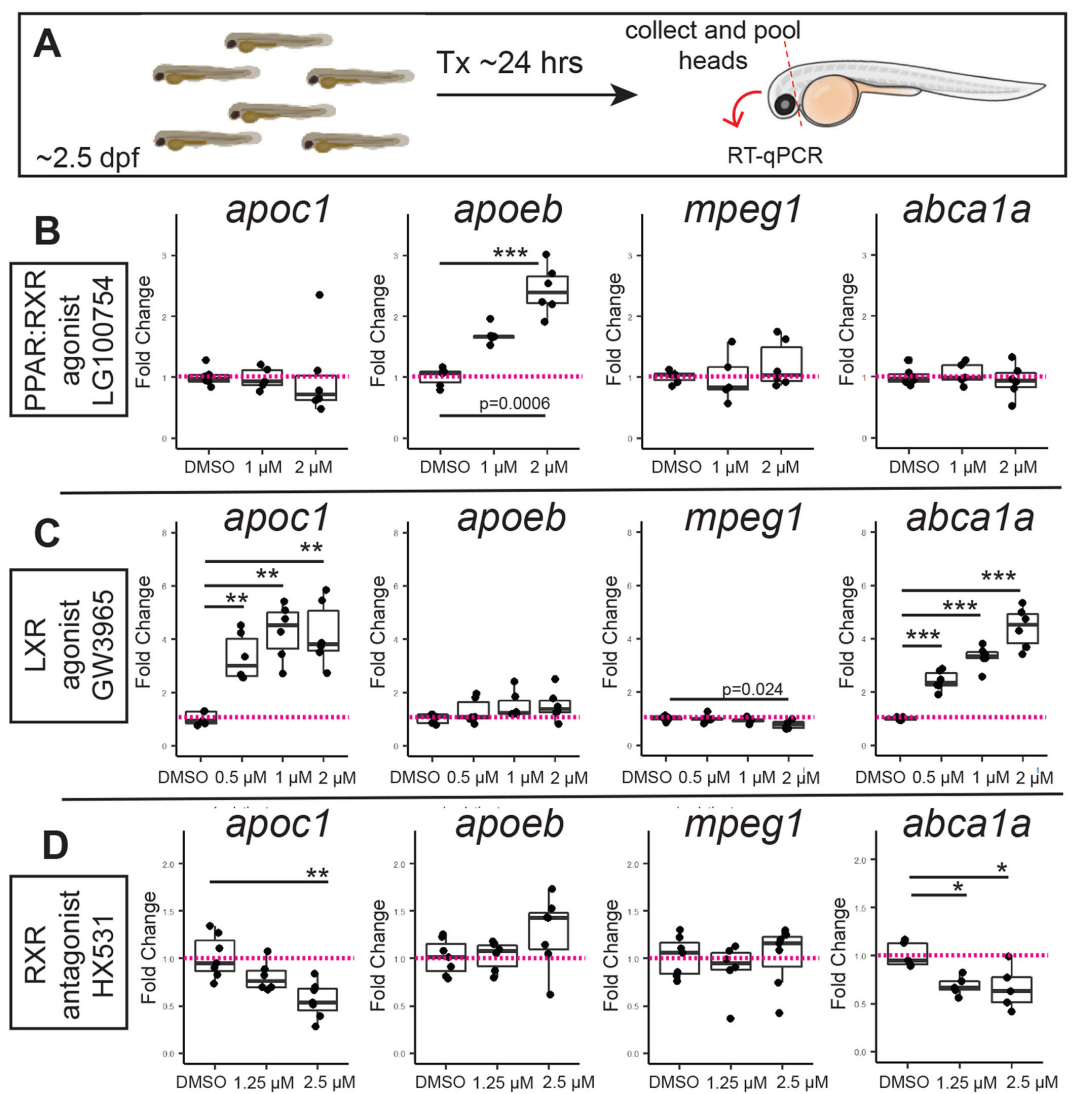
**Effects of pharmacological PPAR, LXR, and RXR modulation on *apoeb* and *apoc1* expression in the developing zebrafish CNS.** (A) Experimental design for treatment by immersion with selected compounds. After 24 h of treatment, whole heads were removed and pooled for each group for RNA extraction and cDNA synthesis. (B) Effects of PPAR-RXR agonist LG100754 on the indicated genes measured by qPCR. (C) Effects of LXR agonist GW3965 on the indicated genes measured by qPCR. (D) Effects of RXR antagonist HX531 on the indicated genes measured by qPCR. In all experiments, the vehicle treated (DMSO) group served as control. Each dot represents the value from one sample of pooled heads (3–9 heads pooled per sample). Statistically significant differences are indicated (***P<10^−4^, **P<0.01, *P∼0.02). Image in A was generated in BioRender.

We used a pharmacological approach to investigate the role of RAR, RXR, LXR, and PPAR receptors in *apoc1* and *apoeb* expression during CNS development. To examine gene expression by RT-qPCR in the developing CNS, we extracted RNA from heads of 3 dpf embryos after 24 h of treatment with selected compounds (Fig. 5A). Treatments began at around 52 hpf to allow for regular development, and so that modulation began after microglia are established in the CNS. After treatment, embryos were collected and anesthetized, and the heads were surgically separated from the body, anterior to the heart, to ensure transcript measurements were from the CNS, and not from other regions of the embryo. Isolation and extraction of RNA from heads, combined with our knowledge of cell types expressing *apoc1* and *apoeb* shown above (Fig. 4), raised confidence that using qPCR would reflect gene expression changes in the CNS, including microglia expressed genes. cDNA was synthesized and gene expression was analyzed by qPCR. For each treatment, we included analysis of a control gene known to be regulated by the target receptor of the pharmacological compound and we also analyzed expression of *mpeg1*, a gene expressed by and used to identify zebrafish microglia (Blume et al., 2020; Casano et al., 2016; Mitchell et al., 2019, 2018; Oosterhof et al., 2017; Sieger et al., 2012), as a control to ensure treatments were not affecting all genes expressed by microglia.

Treatment with the RAR-RXR agonist 9-cisRA did not significantly change *apoc1* or *apoeb* expression (Fig. S4), even though transcripts for the positive control *lws1* (Mitchell et al., 2015) were increased with 9-cisRA treatment. Treatment with the RXR agonist Bexarotene, which strongly increased *lws1* (Mitchell et al., 2015), modestly increased *apoc1* and *apoeb* at the highest doses, though the changes were not statistically significant (Fig. S4). We also failed to find evidence of RA signaling in microglia using real-time images of microglia in RARE:YFP;*mpeg1*:mCherry transgenic embryos (Supplemental Movie 1). The RARE:YFP line reports regions of active retinoic acid signaling with YFP (Perz-Edwards et al., 2001). At ∼3 dpf, there is a YFP+ region reported in the ventral retina (Mitchell et al., 2015; Prabhudesai et al., 2005). Although microglia (*mpeg1*:mCherry+) migrate through the active retinoic acid signaling domains of the ventral retina, the microglia did not show expression of the RARE:YFP reporter (Supplemental Movie 1). Collectively, these results indicate that retinoic acid is not likely a significant regulator of *apoc1* or *apoeb* expression by microglia during early CNS development.

We next investigated effects of the PPAR-RXR agonist LG100754 on *apoc1* and *apoeb* expression. LG100754 is selective for PPAR-RXR heterodimers with strongest effects on PPARγ-RXR and no activity with LXR receptors (Germain et al., 2006; Lala et al., 1996; Schulman et al., 1997). *Apoe* is a reported target of PPAR-RXR transcriptional activity (Galetto et al., 2001), and as expected, LG100754 treatment increased expression of *apoeb* approximately two- to threefold at the highest dose used (Fig. 5B). In contrast, *apoc1* transcripts were not significantly changed in whole heads following LG100754 treatment (Fig. 5B). Interestingly, and somewhat surprisingly, *abca1a* was also not significantly upregulated by LG100754 treatment (Fig. 5B). This suggests that reported PPAR regulation of *Abca1* (Chawla et al., 2001; Chinetti et al., 2001; Ogata et al., 2009) could differ amongst cell types or species, though it is also worth noting that the reported induction of *Abca1* by PPAR receptors may involve PPAR-mediated induction of LXR (Kamijo et al., 2012; Ogata et al., 2009).

We then examined agonism of LXR receptors using the compound GW3965, which is highly selective for LXR and does not show significant activity at other RXR heterodimer partners, including PPAR and RAR (Collins et al., 2002). GW3965 has previously been shown to activate zebrafish LXR (Archer et al., 2008; Reschly et al., 2008). In contrast to PPAR-RXR agonism, treatment with the LXR agonist GW3965 (Collins et al., 2002; Joseph et al., 2002; Zelcer et al., 2007) upregulated *apoc1* transcripts approximately three- to fivefold in whole heads, along with expected increase in the positive control gene *abca1a* (Fig. 5C), which has been shown to be regulated by LXRs (Joseph et al., 2002; Repa et al., 2000). However, GW3965 treatment did not significantly change *apoeb* expression in heads, even at the highest dose. In addition, the highest concentration of GW3965 slightly decreased *mpeg1* transcript levels (Fig. 5C).

Since both PPAR and LXR heterodimerize with RXRs, we used the RXR-specific antagonist HX531 (Ebisawa et al., 1999) as a loss-of-function approach to determining if RXR-dimeric receptors are involved in endogenous induction of *apoc1* or *apoeb* during CNS development. The RXR antagonist HX531 can specifically inhibit RXR heterodimeric receptors of various types (Ebisawa et al., 1999; Kanayasu-Toyoda et al., 2005; Vivat et al., 1997). We found that HX531 decreased *abca1a* transcripts, consistent with LXR-RXR regulation of this gene (Joseph et al., 2002; Lalloyer et al., 2009; Repa et al., 2000) (Fig. 5D). HX531 also decreased *apoc1* but did not change *apoeb* levels (Fig. 5D), suggesting there could be an endogenous role for RXR receptors in the induction of *apoc1* expression by microglia during CNS development.

### Effects of PPAR, LXR, and RXR modulation on apolipoprotein transcript levels expressed by individual microglia

Changes in *apoc1* and *apoeb* transcripts measured by qPCR in whole heads in response to the selected modulators could be due to changes in transcripts in individual cells, changes in the overall number of microglia, or the onset of *apoc1* or *apoeb* expression in cell types other than microglia. To determine which was the case, we used HCR WISH to visualize microglia (*mpeg1*+) in combination with *apoc1* and *apoeb* in the embryonic CNS, following treatments with the selected compounds described above. For these experiments, we chose to use GW3965 (LXR agonist) at 1 µM, LG100754 (PPAR-RXR agonist) at 1 µM, and HX531 (RXR antagonist) at 2.5 µM, because these doses resulted in gene expression changes measured by qPCR for *apoc1* or *apoeb* without affecting *mpeg1* (Fig. 5) and did not show effects on embryo viability. For this analysis, we focused on the eye/retina and optic tectum, which have readily discernable boundaries and could be imaged well on our confocal microscope system.

**Fig. 6.**
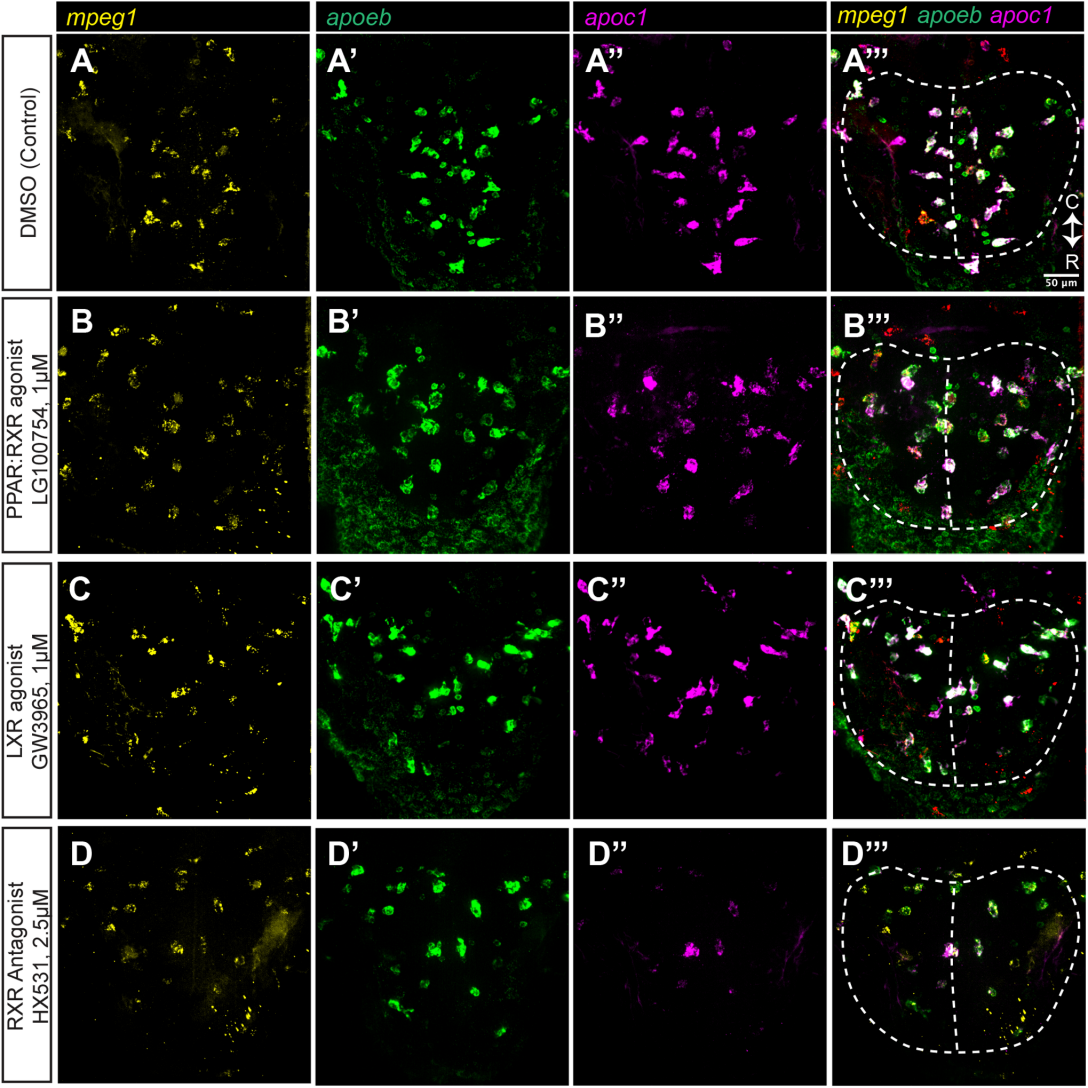
**Expression of *apoc1* and *apoeb* measured in individual microglia in the zebrafish optic tectum by HCR WISH following PPAR, LXR, and RXR modulation.** We performed HCR wholemount *in situ* hybridization (WISH) with probe sets to detect *mpeg1*, *apoc1*, and *apoeb* in embryos following treatment with the indicated compounds. Orientation and regions of imaging are as indicated previously for Fig. 4. (A–D) Single channel and merged images from optic tectum for each treatment group. Orientation markers: C, caudal; R, rostral. (E–H) Merged images of eye/retina. Images are representative of *n*=3–5 embryos per condition.

**Fig. 7.**
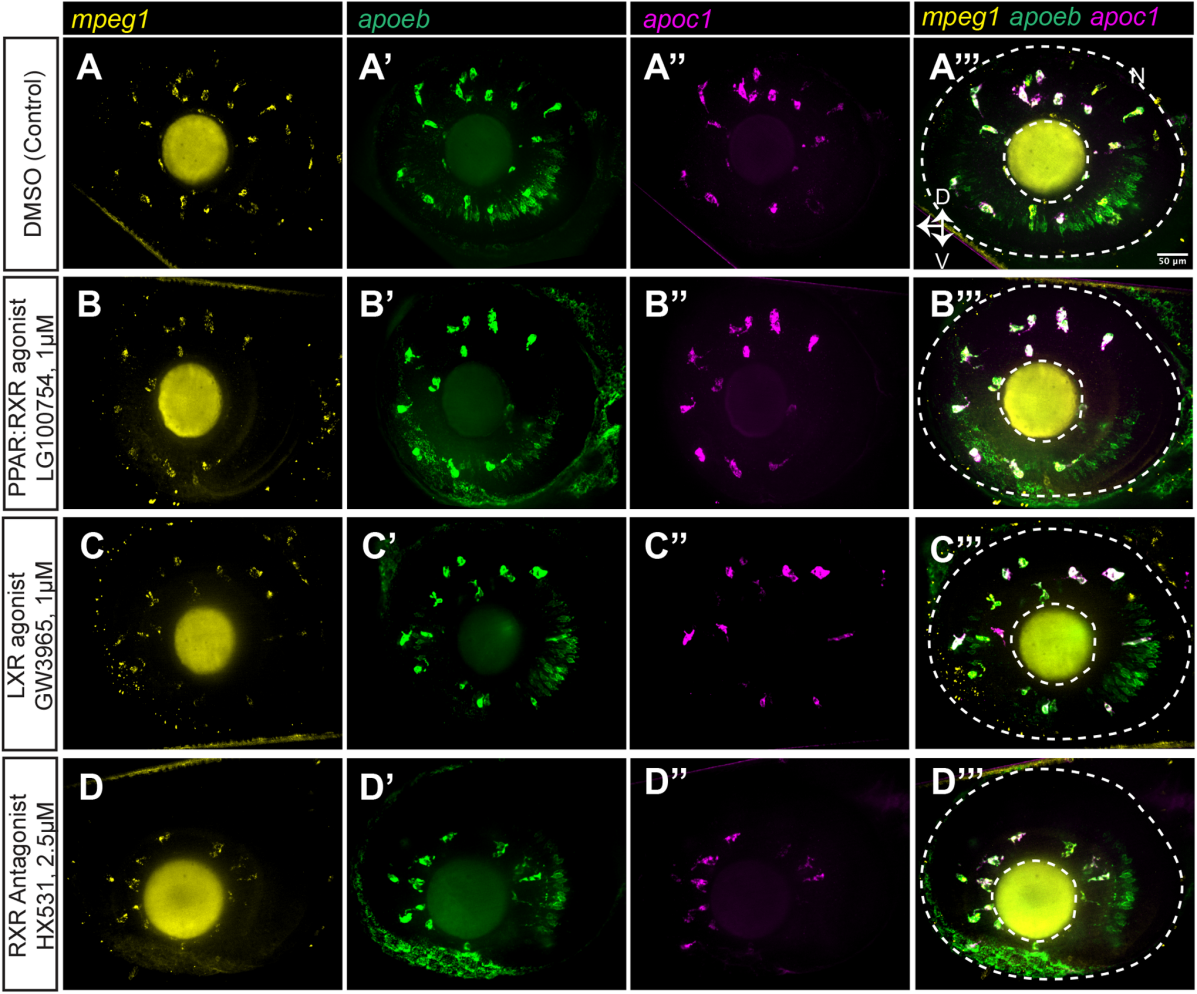
**Expression of *apoc1* and *apoeb* measured in individual microglia in the zebrafish eye/retina by HCR WISH following PPAR, LXR, and RXR modulation.** We performed HCR wholemount *in situ* hybridization (WISH) with probe sets to detect *mpeg1*, *apoc1*, and *apoeb* in embryos following treatment with the indicated compounds. Orientation and regions of imaging are as indicated previously for Fig. 4. (A–D) Single channel and merged images from eye/retina for each treatment group. Orientation markers: D, dorsal; N, nasal; V, ventral. Images are representative of *n*=3–5 embryos per condition.

**Fig. 8.**
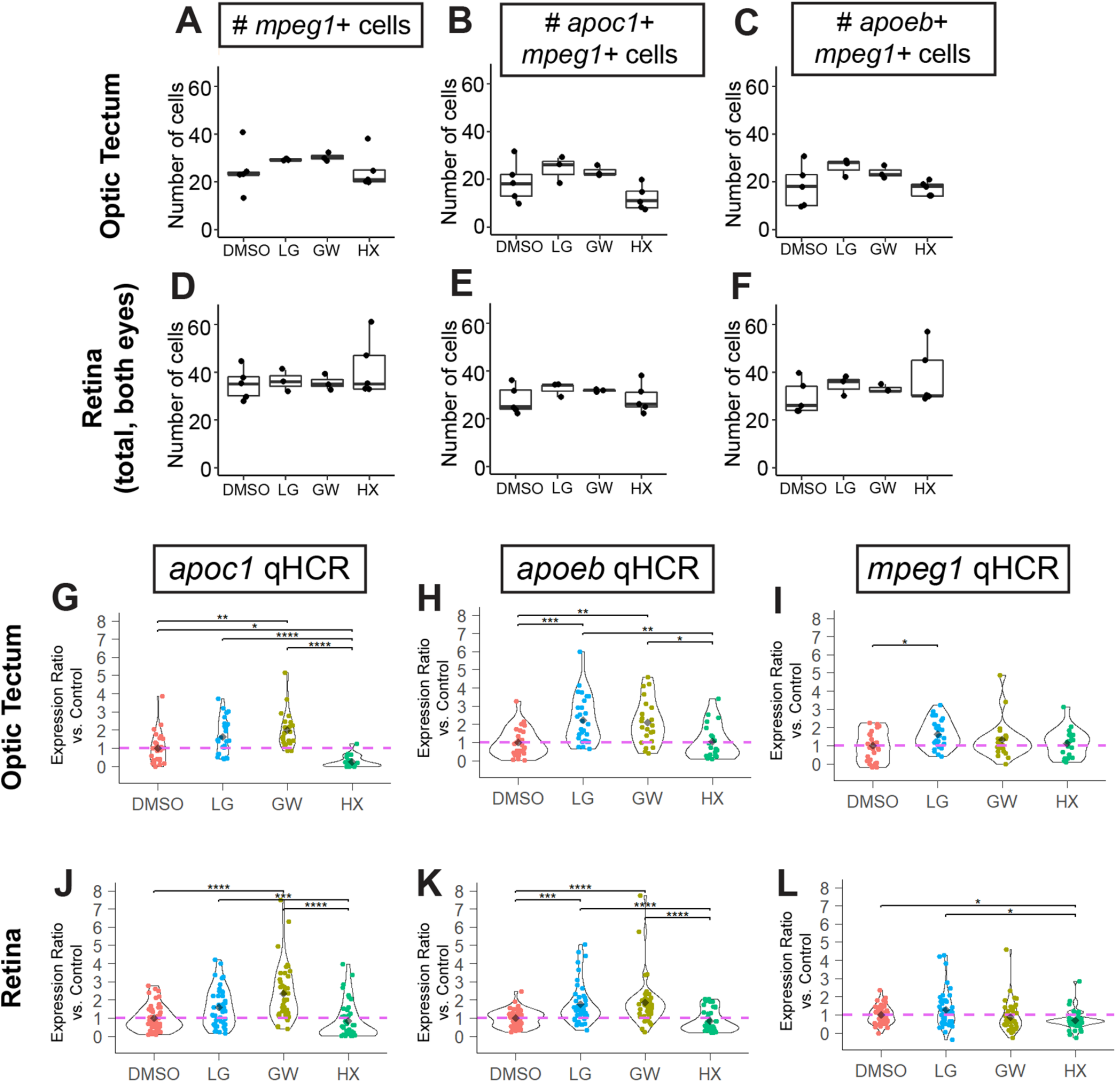
**Quantification of HCR *in situ* images to analyze gene expression in microglia.** Quantification of total numbers of *mpeg1*+ cells in the optic tectum and retina for the various treatments. Each dot represents the count from one embryo (A,D). Quantification of number of *mpeg1*+ cells also expressing the gene of interest, *apoc1* or *apoeb* (B,C,E,F). (G–L) Analysis of signal intensity in individual microglia using qHCR for *apoc1* (G,J), *apoeb* (H,K), and *mpeg1* (I,L) transcripts detected in optic tectum or retina following the indicated treatments. Violin plots represent the distribution of individual cell measurements from each group, shown as a ratio of expression level compared to the average of the control group for each gene of interest. Each colored dot represents the value from one individual microglial cell. The black diamond within the violin plot indicates the mean of the group. Individual cell measurements were obtained from a total of 3–5 embryos per group. Statistically significant differences are indicated (****P<10^−4^, ***P<0.001, **P<0.01, *P<0.05).

Visual inspection of the HCR probe signals for each of these transcripts in the CNS of embryos treated with LG100754, GW3965, and HX531 show similar patterns to that of control embryos, indicating that expression of these genes remains restricted to the same cell types (Figs 6 and 7, optic tectum and eye/retina, respectively). There was only an occasional *apoc1*+ cell that was not also *mpeg1*+, and *apoeb* is expressed by *mpeg1*+ microglia and other cell types as described above (Figs 6 and 7, ring-like staining in the optic tectum and presumably Müller glia in the retina). The rare *apoc1*+*mpeg1*- cells observed displayed morphology suggesting that these cells are also microglia. Treatments with the three compounds did not significantly change the overall number of *mpeg1*+ cells in the optic tectum and retina (Fig. 8A,D), indicating that effects of the treatments cannot be attributed to changes in numbers of microglial cells. Instead, measured differences in transcripts are most likely due to changes transcript levels in microglia on a per cell basis. To first examine this possibility, we scored each individual microglia (*mpeg1*+ cell) in the eye/retina and optic tectum for *apoc1* or *apoeb* expression (positive or negative, based on visible HCR probe signal; Fig. 8B,C,E,F). The number of microglia expressing *apoc1* was not found to be different for GW3965, LG100754, or HX531 treatments compared to controls (Fig. 8B,E). Similarly, the number of microglia expressing *apoeb* was not found to be different for any treatment group compared to controls (Fig. 8C,F). We also analyzed these data as a ratio of *apoc1*-positive microglia out of the total number of *mpeg1*+ microglia, and as a ratio of *apoeb*-positive microglia out of the total number of *mpeg1*+ microglia; there were no statistically significant differences between treatments (not shown). Collectively, counts and ratio results indicate that transcripts for these genes are increased, or decreased, on a per cell basis in response to treatment.

To quantitatively assess expression of these transcripts in individual microglia, we used the quantitative nature of fluorescence from HCR probe sets in which fluorescence intensity scales with transcript abundance (Choi et al., 2018; Trivedi et al., 2018). Individual microglia from brain and optic tectum were traced in raw z-stack images and the corrected total fluorescence per cell was determined from the integrated density (total cell fluorescence) obtained from the microglial cell volume after background signal subtraction. Using this method of analysis, we analyzed transcript levels in response to compound treatments on an individual microglial cell basis (Fig. 8G–L). At the concentration of LG100754 (PPAR-RXR agonist) used in these experiments (1 µM), there was a modest increase in *apoeb* detected by qPCR (Fig. 5B) without significant effects on *apoc1*. Consistent with the qPCR data, treatment with PPAR-RXR agonism increased *apoeb* expression approximately twofold (Fig. 8H,K) with very little effect on *apoc1* (Fig. 8G,J), in individual microglia measured by qHCR. Also consistent with our qPCR data (Fig. 5C), *apoc1* was increased in individual microglia by GW3965 (LXR agonist, 1 µM) treatment (Fig. 8G,J), though this change was less dramatic than measured by qPCR (approximately two- to threefold by qHCR, and approximately two- to fivefold by qPCR). GW3965 also modestly increased *apoeb* expression in individual microglial cells measured by qHCR (∼1.8-fold; Fig. 8H,K). A similar fold increase in *apoeb* in response to GW3965 was measured by qPCR (Fig. 6C) though not found to be statistically significant. Consistent with the qPCR data, RXR antagonism using HX531 treatment reduced *apoc1* expression in individual microglia particularly in the optic tectum (Fig. 8G,J), but did not strongly change expression of *apoeb* by microglia (Fig. 8H,K). None of the treatments resulted in strong changes to *mpeg1* in individual microglia, though HX531 treatment did very slightly decrease levels of *mpeg1* transcripts in some microglia in the retina (Fig. 8I,L).

Overall, the qHCR results (measured from individual microglia) are mostly consistent with the qPCR results (measured from whole homogenized heads), with additional insight into differential regulation of these genes in individual microglia within the retina and optic tectum, that was not apparent from homogenized tissue measurements. Of note, decreases in *apoc1* transcript levels induced by RXR antagonism were more significant in microglia within the optic tectum versus the retina, decreasing *apoc1* to ∼0.26-fold in the optic tectum and to ∼0.8-fold in the retina (Fig. 8G,J). The individual qHCR cell measurements also show that some cells dramatically respond to each treatment, while others have a more modest change in gene expression. This is notable, for example, in that some retinal microglia increase *apoc1* or *apoeb* as much as seven- to eightfold in response to GW3965 treatments (Fig. 8J,K). It is also worth noting that statistical significance was found for changes in some genes measured by qHCR that were not found when measured by qPCR. In addition, some of the fold changes measured by qPCR were more dramatic than that measured by qHCR, for example, *apoc1* upregulation in response to GW3965 treatment. There are several likely explanations for such apparent discrepancies which we outline in the discussion section. Collectively, the cell counts and measurement of transcript levels by qPCR and qHCR indicate that modulation of LXR-RXR receptor signaling impacts *apoc1* expression in individual microglia, both by increasing expression through agonism and decreasing expression through antagonism. In contrast, the data suggest that agonism of both LXR and PPAR-RXR modestly increase *apoeb* expression by microglia, but RXR antagonism does not have significant effects on microglial expression of *apoeb*.

### Expression of lxra (*nr1h3*) and pparg (*nr1c3*) by microglia

**Fig. 9.**
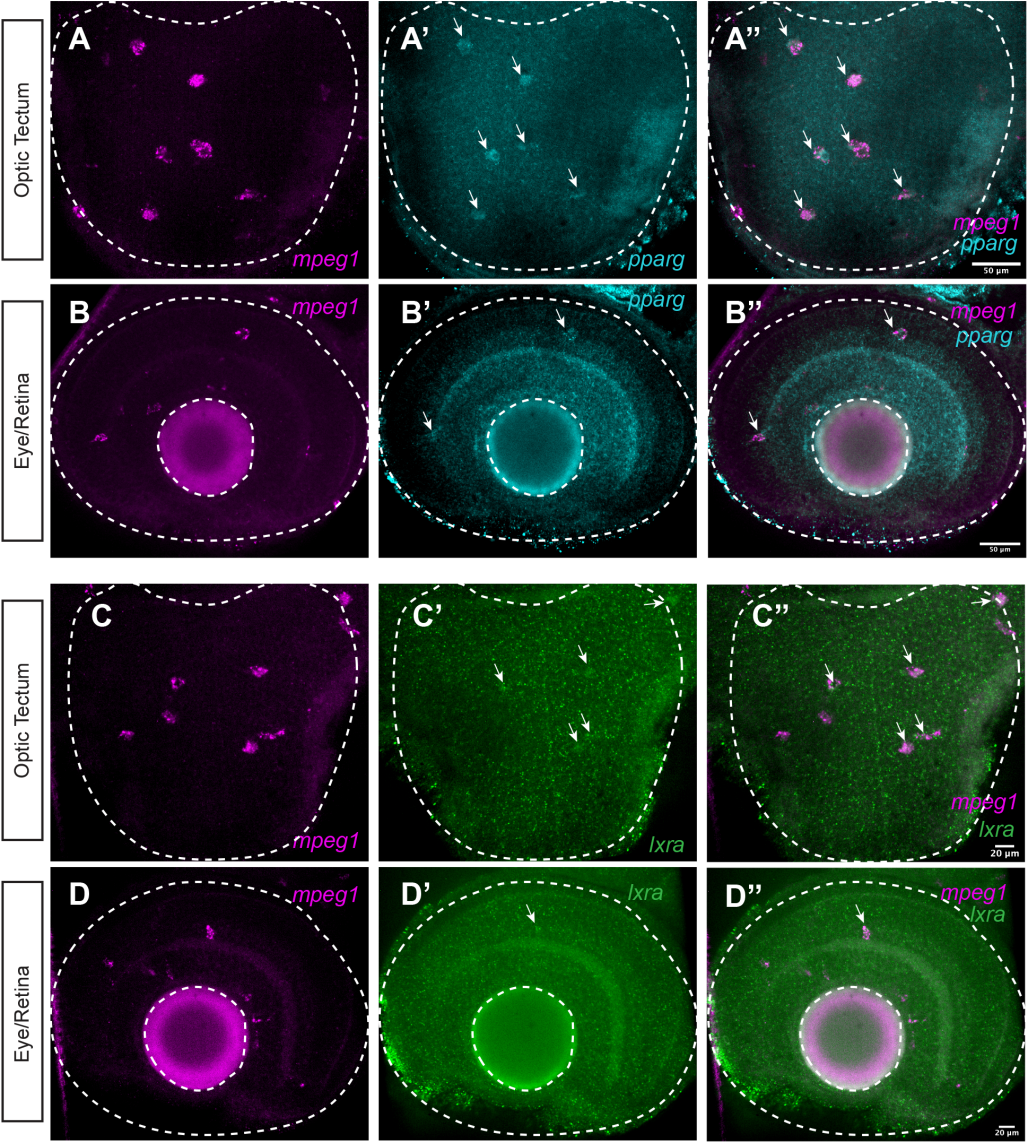
**Expression of *nr1c3 (pparg)* and *nr1h3 (lxra)* by microglia in the developing CNS.** We performed HCR WISH with probe sets to detect *mpeg1*, *nr1c3 (pparg)*, and *nr1h3 (lxra)* in the CNS of 3-day-old zebrafish embryos (A–D). Single channel and merged images from optic tectum (A–B″) and eye/retina (C–D″) are shown. Images are z projections from 2–3 z stacks taken at 1 micron step size. Arrows indicate signal from *pparg* probe or *lxra* probe that co-localizes with *mpeg1* probe signal. Images are representative of *n*=5 embryos.

The measured effects of pharmacological agonism of LXR-RXR and PPAR-RXR receptors and antagonism of RXR receptors could be due to direct or indirect effects of these compounds by modulating expression directly in microglia, or indirectly through other cell types that express these receptors then act on microglia in some way. We therefore examined expression of LXR and PPAR receptors at the mRNA level in microglia using multiplex HCR *in situ* hybridization, with probe sets to detect *mpeg1*, *nr1c3* (*pparg*), and *nr1h3* (*lxra*) (Fig. 9), to determine if microglia could have the ability to directly respond to pharmacological treatments. We focused on these receptors because (i) they function as heterodimers with RXRs, (ii) zebrafish have only one LXR receptor gene (*nr1h3*) (Schaaf, 2017), (iii) the LG100754 compound is most selective for PPARγ-RXR heterodimers (Germain et al., 2006; Lala et al., 1996; Schulman et al., 1997) indicating that effects are most likely due to PPARγ modulation, (iv) there are a number of RXR receptor isoforms with little information about the heterodimer pairing in zebrafish, and (v) because genes for RXR receptors have been expanded in zebrafish due to the teleost genome duplication. Transcripts for *nr1c3* (*pparg*) co-localized with *mpeg1* in the developing zebrafish CNS (Fig. 9), indicating that microglia do indeed express PPARγ. We also detected weak expression of *nr1h3* (*lxra*) in *mpeg1*+ cells, which was more apparent in the optic tectum (Fig. 9C–C″) than the developing retina (Fig. 9D–D″). The expression of both of these nuclear hormone receptors indicates that microglia could directly respond to LXR and PPAR agonism by modulating expression of *apoc1* and *apoeb*. Although expression of *pparg* and *lxra* transcripts is apparently at low levels, it is worth considering that only minimal amounts of these receptors could be needed to modulate their target genes. Additionally, given the staining pattern of the *nr1h3* (*lxra*) probes, we cannot exclude the possibility that other cells in the CNS, or even in the embryo body, could respond to LXR agonism then indirectly induce a response in microglia. Likewise, we also cannot exclude the possibility of indirect effects of PPARγ agonism and RXR antagonism on microglial apolipoprotein expression.

## DISCUSSION

In this work, we demonstrate and characterize microglial expression of apolipoprotein transcripts *apoc1* and *apoeb in situ* in the zebrafish. We show that *apoeb* localizes to microglia as well as other cell types in the brain and retina, while *apoc1* expression is unique to microglia. In addition, we provide evidence that other macrophages in the early embryo do not express *apoc1*, suggesting unique factors induce microglial expression of this gene. Our work also reveals differential regulation of microglial expression of *apoc1* and *apoeb* in zebrafish, through modulation of RXR receptors. We found that microglia express transcripts for both LXR and PPARγ, indicating that a direct response to pharmacological modulation of these receptors is possible. Though we did detect expression of LXR and PPAR receptor transcripts in microglia in the developing CNS, we acknowledge that our results cannot distinguish between direct or indirect effects of RXR heterodimer modulation, or other levels of control of gene expression such as transcript stability, and that possible off-target effects of these compounds could exist. The effects of RXR antagonism suggests that there could be an endogenous function of RXR receptors in the induction of *apoc1* during early microglial colonization of the zebrafish CNS, though genetic manipulations are required to determine if this is the case.

In comparison of qPCR (Fig. 5) and qHCR analyses (Fig. 8G–L), we noticed some discrepancies in the magnitude of the fold change measured. It is possible that microglial cells outside of the tissues selected for qHCR analysis may have more dramatic changes in gene expression, that there are differences in sensitivity of qPCR and qHCR within certain ranges of transcript abundance, differences in statistical power of per cell versus bulk tissue measurements/sample sizes, the relative quantification of qPCR versus signal intensity in qHCR, and/or that other cell types in the head that we did not directly image or analyze could contribute to overall changes measured by qPCR from homogenized whole heads. The former is likely most influential for *apoeb* measurements, given that this gene is also expressed by other cell types besides microglia, and these transcriptional changes would be measured in bulk qPCR samples. Nonetheless and overall, similar trends were observed in both analyses.

While we demonstrate expression of *apoc1* is unique to microglia in the developing zebrafish CNS and adult retina; such restricted expression in the CNS of other vertebrates remains to be determined. It is possible that expression of this gene could be different *in vivo* versus *in vitro*, in different species, at different timepoints of development, or between tissues (such as brain versus retina). Regardless, the function of APOC1 in the CNS remains unknown. Studies in mouse models indicate *Apoc1* may have effects on cognitive function (Abildayeva et al., 2008; Berbée et al., 2011), but the mechanisms by which this occurs, or whether it could be related to changes in the CNS versus peripheral lipid metabolism, are not clear, especially given uncertainties about expression of this gene by mouse microglia (Gosselin et al., 2017). APOE has been shown to regulate uptake and metabolism of amyloid-ß (Castellano et al., 2011; Kim et al., 2009; Verghese et al., 2013), but whether APOC1 has a similar role is not known. In the periphery, apolipoproteins are an integral component of particles that transport lipids and cholesterol for metabolic regulation via the liver. Both APOE and APOC1 are components of human high-density lipoproteins, HDLs (Cudaback et al., 2012; Fuior and Gafencu, 2019). It is likely that APOC1 and APOE also have a role in lipid/cholesterol transport in the CNS (Wang and Eckel, 2014; Yin et al., 2021), and this could be particularly crucial for synaptic maintenance and function given that neuronal synapses are cholesterol rich with rapid turnover. Further, due to regulated blood-CNS permeability and transport, maintaining cholesterol and lipid homeostasis in the CNS could be achieved via various pathways including those using recycling and/or efflux, and may be dependent on glial cells (Mauch et al., 2001; Tracey et al., 2018; Yin et al., 2021).

Previous reports have suggested that genes in the apolipoprotein cluster are coordinately regulated (Cudaback et al., 2012; Evangelou et al., 2019; Mak et al., 2002). However, our results suggest that PPAR-RXR and LXR-RXR receptors can differentially regulate *apoc1* and *apoeb* in zebrafish microglia. While PPAR-RXR agonism increased *apoeb*, it did not have strong effects on microglial expression of *apoc1*. In addition, LXR agonism strongly increased *apoc1*, with only very modest increase in *apoeb* expression by microglia. Previous reports have concluded that LXRs regulate APOE in mouse macrophages (Laffitte et al., 2001b) and in astrocytes (Liang et al., 2004) and we also measured modest LXR-agonist increases in *apoeb* in zebrafish microglia on an individual cell basis. However, RXR antagonism had significant effects on microglial expression of *apoc1* with minimal effects on *apoeb*, suggesting that RXR receptor signaling could possibly be an endogenous mechanism by which microglia obtain high levels of *apoc1* transcripts. Given that microglia express transcripts for LXR, we speculate that this induction could occur via LXR-RXR, and that ligands for both LXR and RXR could contribute to expression of this gene. Our findings also suggest that RXR receptors are not significant drivers of *apoeb* expression by microglia during development, though stimulation of PPAR-RXR could serve to increase expression of *apoeb*. In addition, it remains to be determined if the effects of these modulators are similar or different in adult animals compared to that of development.

In previous work, RXR permissivity with LXR receptors was reported (Lalloyer et al., 2009). We measured modest upregulation of *apoc1* with RXR agonism, though it was comparatively lower and more variable to that of LXR agonism and was not statistically significant. It is possible that RXR antagonism could affect PPAR or LXR heterodimers, or potentially even RXR homodimers. Regardless, our results support an endogenous role for RXR receptors in inducing expression of *apoc1* in microglia. Given the agonist treatment results, we hypothesize that this induction occurs LXR-RXR heterodimeric receptors, though we cannot exclude the possibility that other RXR receptors are involved. It is worth noting that although an LXRα ortholog exists, there is no known LXRβ ortholog in zebrafish (Schaaf, 2017). While we did not find any predicted LXR factor binding sites in our analysis of the 5 kb region upstream of the *apoc1* transcription start site, other work shows LXR ligands affect *APOC1* transcription in macrophages (Mak et al., 2002), and we were able to detect transcripts for *lxra* (*nr1h3*) in microglia (Fig. 9). We may not have identified LXR binding sites because they exist outside of the region analyzed, the effects of LXR agonism are indirect, or because the analysis tool (which is based on human consensus transcription factor binding sequences) could not identify predicted zebrafish LXR binding sites; the LXR consensus sequences for zebrafish, to our knowledge, have not been described.

Lipids are known endogenous PPAR-RXR ligands and known endogenous LXR-RXR ligands include cholesterol derivatives such as oxysterols. Given the well-appreciated phagocytic activity of microglia in the CNS in engulfment and degradation of apoptotic cells in developmental and homeostatic contexts, it is tempting to speculate that regulation of these apolipoprotein genes in microglia could be linked to lipids and cholesterol obtained upon apoptotic cell engulfment. This would provide a mechanism for microglia to sense and respond to metabolic changes in the CNS environment. In support of this idea, LXR transcriptional activity in macrophages (A-Gonzalez et al., 2009), as well as cholesterol efflux mechanisms (Kiss et al., 2006), appear to be stimulated downstream of apoptotic cell recognition. Such a link is worth exploring in future experiments.

The differential regulation of microglial expression of *apoeb* and *apoc1* in response to PPAR and LXR agonists in zebrafish, respectively, and to that of RXR antagonism, indicates that these genes could be differentially targeted in therapeutic approaches to treat human disease, if that were so desirable. The impacts of such selective modulation would depend on future studies to understand if one gene or the other may differentially contribute to or alleviate disease, which remains to be determined. Of note, LXR agonists have been appreciated for their therapeutic potential in a variety of contexts, including neurodegenerative disease (Fitz et al., 2019; Zelcer et al., 2007). It remains to be determined if differential regulation of *APOE* and *APOC1* by these receptors is conserved in humans, as it has been noted that differences in apolipoprotein gene regulation by PPAR versus LXR may differ between mouse and human macrophages (Laffitte et al., 2001a). This suggests that in addition to cell type differences, there may be species-specific differences in regulation of these genes. Interestingly, the RXR agonist Bexarotene was pursued as a treatment for AD (Tousi, 2015), partly due to reported ability to increase APOE expression, but this has been reconsidered (Balducci et al., 2015; Tousi, 2015). It is worth considering how responses could differ by cell types, between species, or for developmental stages. Indeed, a better understanding of effects of RXR receptor modulation in the CNS is necessary.

Collectively, our work supports the use of the zebrafish to better understand the regulation and function of these disease-associated apolipoproteins in the CNS. Such work will contribute to a better understanding of homeostatic functions that are disrupted in neurodegenerative diseases, and the role of microglia in such contexts.

## MATERIALS AND METHODS

### Animals

All procedures using zebrafish were performed in compliance with IACUC (Institutional Animal Care and Use Committee) approved protocols at the University of Idaho. Adult zebrafish (*Danio rerio*) were maintained on a 14:10 h light:dark cycle in 28.5°C recirculating, monitored system water, and were housed and propagated following Westerfield (2007). Zebrafish lines used in this work include a wild-type strain, referred to as SciH, originally obtained from Scientific Hatcheries (now Aquatica Tropicals), *mpeg1*:mCherry (Ellett et al., 2011) (originally obtained from Zebrafish International Resource Center, ZIRC), and RARE:YFP (Perz-Edwards et al., 2001) (obtained from Dr Deborah Stenkamp's stock at University of Idaho; originally gifted to Dr Deborah Stenkamp by Dr Elwood Linney). Embryos were collected into glass beakers in the morning, with light onset considered to be 0 h post fertilization (hpf), and water was refreshed daily until experimental endpoints. In experiments using microscopy of whole embryos, phenothiourea (PTU, 0.003% final concentration) was added to the fish water to prevent pigment development. Zebrafish cannot be sexed before reproductive maturity and so could not be determined for experiments involving embryonic zebrafish; adult zebrafish of both sexes were used for collection of adult retinal tissue.

### RNA extraction from whole retinas, cDNA synthesis, PCR, and apoc1 cDNA cloning

Following dark adaption, whole eyes were enucleated from one year male and female SciH adult zebrafish. Retinas were dissected from eyes as described (Mitchell et al., 2018) and the retinal pigmented epithelium (RPE) was removed. Retinas were submerged in RNA lysis buffer (Machery-Nagel RNA extraction kit) and homogenized using a pellet pestle. RNA was extracted using an RNA extraction kit (Machery-Nagel), following the manufacturer's protocol. A Nanodrop^®^ ND-1000 Spectrophotometer was used to check RNA yield and quality. Synthesis of cDNA was performed using SuperScript^®^ IV Reverse Transcriptase kit (Invitrogen), with random hexamer and oligodT primers.

Three separate primer pairs (Table 1) were used to amplify selected *apoc1* cDNA sequences. Primers were designed based on the Ensembl database, using the zebrafish genome build 11 (GrcZ11). PCR reactions were performed using Q5 polymerase Master Mix (NEB). A volume of 1μL of adult retinal cDNA was used as template, and the manufacturer's recommended cycling conditions were used. PCR products were transferred to gel electrophoresis [2% agarose, with TAE (Tris base, acetic acid, EDTA) Buffer] and imaged using a Bio-Rad Gel Doc-1000 and Quantity One imaging software. Subsequently, using a blue light box (Clare Chemical Research Dark Reader^®^ Transilluminator), bands were excised from the agarose gel then extracted using NEB Monarch Gel Extraction kit. To increase product yield, the extracted PCR products were used as templates in a second PCR re-amplification reaction using the same primer pairs. The re-amplified PCR products were then again run on an agarose gel and extracted.

**Table 1.**
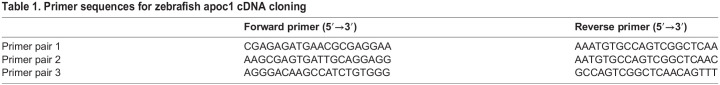
Primer sequences for zebrafish apoc1 cDNA cloning

Purified PCR products were ligated into the pMiniT vector using the NEB^®^ PCR cloning kit, following the manufacturer's instructions. A ratio of 3:1 (insert:vector) was used. The ligation product was transformed into NEB 10-beta *E. coli* competent cells following the manufacturer's protocol, and transformants were plated and grown on LB-Amp plates. Single colonies were then selected to inoculate liquid cultures. Plasmids were extracted using QIAprep^®^ Spin Miniprep kit (Qiagen). Plasmids were screened for successful ligation using restriction enzyme digestion. Plasmids containing inserts of correct size were verified by Sanger Sequencing using the Cloning Analysis Primers provided by the NEB cloning kit. Sanger Sequencing was performed at Washington State University for Reproductive Biology Core (WSU CRB). Sequences mapped to the expected exons 1-4 of *apoc1*, which included the 5′ and 3′ UTR, and excluding the intronic regions. Of the three primer pairs, the cDNA product from primer pair 2 (99% ID to *Danio rerio apoc1* mRNA consensus sequence; Fig. S2) was selected for use as a template to generate RNA probes.

### Generation of DIG-labeled RNA probes

Purified plasmid containing *apoc1* cDNA insert (primer pair 2 product) was linearized with PacI or BamHI, then precipitated with 1.5 volume of cold 100% Ethanol, and stored at −80°C for 2 h or overnight. Linearized plasmids were then spun twice at 14,000×***g*** for 10 min, with one rinse in 1 ml 70% Ethanol in between spins. The supernatant was decanted, and the pellet was air dried, then resuspended in 50 μl of RNAse-free water. 1 μg of the linearized template was used for *in vitro* RNA transcription, using either T7 or SP6 polymerase (to generate both sense and anti-sense probes), and DIG-labeled RNA nucleotides using the DIG RNA Labeling Kit (SP6/T7; Millipore-Sigma). At the end of the reaction, tubes were spun twice for 15 min at 14,000×***g*** with one rinse with 70% Ethanol in between. Supernatant was then decanted, and the RNA pellet was air dried, then resuspended in 50 μl RNAse-free water. Probe concentration was measured using a Nanodrop^®^ ND-1000 Spectrophotometer, then aliquoted and stored at −20°C until use.

### *In situ* hybridization of fixed tissue using DIG-labeled RNA probes

Whole retinas were dissected from 6-month-old *mpeg1*:mCherry transgenic (Ellett et al., 2011) fish using the same protocol described above. The retinas were fixed in a 4% PFA in 1X PBS RNAse-free solution overnight at 4°C, washed in 100% methanol, and stored at −20°C in 100% methanol. The *in situ* hybridizations were carried out as previously described (Stevens et al., 2011). In brief, the tissue was rehydrated in a decreasing concentration series of methanol, treated with 10 μg/ml proteinase K for 30 min, and hybridized overnight at 56°C with 1 mg/ml probe in probe hybridization solution. An anti-DIG-POD antibody (Millipore-Sigma), followed by tyramide signal amplification with a Fluorescein fluorophore (Perkin Elmer^®^ TSA™ kit), was used to amplify the probe hybridization signal for fluorescent detection.

At 3 and 5 dpf, *mpeg1*:mCherry zebrafish embryos were anesthetized and fixed in a 4% PFA in 1X PBS RNAse-free solution overnight at 4°C, washed in 100% methanol, and stored at −20°C in 100% methanol. The *in situ* hybridizations were carried out as described before for the whole retina protocol with minor changes. Dehydration in 100% methanol and a Xylene wash were included before the rehydration in order to clear the pigment accumulated at this stage in development. The proteinase K treatments were also shortened to 10 min for the 3 dpf embryos and 20 min for the 5 dpf embryos.

### Immunolabeling of whole fixed tissue

Due to degradation of the transgenic fluorescent signal during the *in situ* procedure, immunolabeling was performed to detect the mCherry protein. After the *in situs* were performed the tissue was washed with PBST (phosphate buffered saline, 0.1% Tween) then placed into antibody dilution buffer over night at 4°C with agitation. Following another wash in PBST, the tissue was placed into primary antibody solution containing rabbit anti-mCherry at 1:100 dilution (Genetex) and DAPI (Thermo Fisher Scientific) overnight at 4°C with agitation. The tissue was washed in PBST and placed into secondary antibody solution containing donkey anti-rabbit Cy3 antibody (Jackson Immunoresearch) overnight at 4°C with agitation. Tissue was then washed again in PBST and received a final wash in 1X PBS (phosphate buffered saline) before storage in 1X PBS at 4°C until imaging.

### *In silico* transcription factor binding site prediction

We used the PROMO virtual laboratory (Farré et al., 2003; Messeguer et al., 2002) tool to analyze the 5 kb region upstream of the *apoc1* transcription start site. We used the PROMO website (http://alggen.lsi.upc.es/cgi-bin/promo_v3/promo/promoinit.cgi?dirDB=TF_8.3), selected ‘human factors’ to search for human consensus binding sites, and entered the 5 kb sequence corresponding to the 5 kb upstream of the transcription start site of *apoc1;* sequence obtained from www.ensembl.org. The dissimilarity rate cut-off was selected at 15%. We selected human factors for this analysis due to zebrafish transcription factors not being as comparatively well annotated, and initial results using zebrafish transcription factor predictions were minimally informative.

### Pharmacological treatments

Stock solutions of Bexarotene (Millipore-Sigma), 9-cis retinoic acid (9-cis RA; Millipore-Sigma) GW3965 (Tocris), LG100754 (Tocris), HX531 (Tocris) were prepared in dimethylsulfoxide (DMSO; Millipore-Sigma), aliquoted, and stored in the dark at −20°C. The 9-cis RA was also stored under nitrogen. Embryos were collected, reared, and treated as described previously (Mitchell et al., 2015) with treatments performed at 28.5°C, beginning at around 52 hpf, and lasting 24 h total. Once the 24-h treatment was complete, embryos were anesthetized, and the heads were surgically separated from the body, anterior to the heart, using dissecting scissors to ensure transcript measurements were from the CNS, and not the yolk sac or region of the developing liver. Any treatment groups showing gross morphology defects and viability affects were noted. At the doses used, the highest dose of LG100754 (2 µM) occasionally affected viability of a few embryos. Heads were immediately transferred to RNA Later (Invitrogen). Heads from three to nine embryos from each treatment group were pooled into RNA Later and stored at −20°C until RNA extraction.

### RNA extraction from heads and cDNA synthesis

To prepare for RNA extraction, samples were brought to room temperature, RNA Later was pipetted off, and then samples were diluted with RNAse-Free water prior to the homogenization and lysis step. RNA extraction was performed using NucleoSpin^®^ RNA Kit (Machery-Nagel) following kit instructions. Samples were homogenized after the addition of Buffer RA1 and β-mercaptoethanol. Kit instructions were followed for RNA purification from cultured cells and tissues beginning at filtrate lysate step. DNA digestion with DNAse was performed as indicated by the manufacturer. Samples were eluted in RNAse-Free water. A nano spectrophotometer (NanoDrop ND-1000 Spectrophotometer) was used to determine yield and purity of RNA samples. In some cases, where A260/A230 ratios were low, an additional clean-up was performed by precipitation with 5 M Ammonium Acetate. After air drying, the pellet was resuspended in 20 µl of RNAse-Free water. Yield and purity were rechecked using nano spectrophotometer. RNA samples were stored at −80°C until cDNA synthesis.

cDNA was synthesized using Superscript IV First-Strand cDNA Synthesis Reaction kit (Invitrogen), following the manufacturer's instructions, using random hexamer primers, and the same amount of RNA input was used for samples from the same experiment (120–495 ng, depending on the yield from samples within an experiment). The final concentration of cDNA was calculated based on RNA input. cDNA samples were stored at −20°C until qPCR reaction set up.

### Quantitative reverse transcriptase polymerase chain reaction (qRT-PCR or qPCR)

Amplification was performed on an Applied Biosystems Step OnePlus Real-Time PCR System using Power Tracker SYBR Green Master Mix (Applied Biosystems) and exon spanning, transcript specific primer pairs (Table 2). A cDNA input of 5 ng was used for all qPCR reactions. Relative quantification of gene expression between control and treated samples was determined using the calibrator genes *beta-actin2* and *18s*, and the 2^ddCt method using the geometric mean of the two control genes.

**Table 2.**
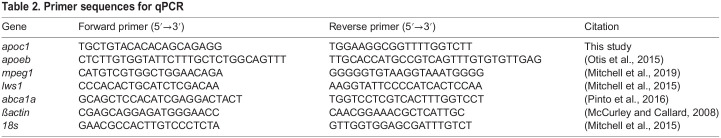
Primer sequences for qPCR

### *In situ* hybridization with whole embryos using HCR

Probe sets and hairpins to detect transcripts of interest using HCR *in situ* hybridization were purchased from Molecular Instruments (Choi et al., 2018). Probe set size and NCBI accession number used for design are shown in Table 3. At the timepoint of collection, embryos were fixed in freshly made 4% PFA in phosphate buffered saline (1X PBS) at 4C for 24 h. The following day, embryos were washed three times in 1X PBS then dehydrated and permeabilized in 100% Methanol washes (four 10-min washes, followed by one 50-min wash), then stored in fresh 100% Methanol at −20°C for at least 1 day, and up to 2 months. To prepare for HCR *in situ* hybridization, embryos were rehydrated with a graded series of methanol and phosphate-buffered saline solution containing 0.1% Tween-20 (PBST). The graded series consisted of the following washes for 5 min each at room temperature: 75% methanol:25% PBST, 50% methanol:50% PBST, 25% methanol:75% PBST, and 100% PBST. Next, embryos were treated with proteinase K (10 µg/ml) for 10 min at 37°C, washed with PBST, then post-fixed with 4% PFA in 1X PBS at room temperature for 20 min. Then, embryos were washed again with PBST before entering the detection stage. The following steps were performed in an Enviro-Genie hybridization oven (Scientific Industries) with constant rocking. Embryos were pre-hybridized using probe hybridization buffer (Molecular Instruments) for 30 min at 37°C. While incubating, the probe solution was prepared by adding 2 pmol of each probe set (*mpeg1*-B1, *apoc1*-B2, and *apoeb*-B3; Molecular Instruments) to 500 µl of probe hybridization buffer. After incubation, the pre-hybridization solution was removed, and embryos were immersed in the probe solution and incubated for 12–16 h at 37°C. The next day, excess probe solution was removed by washing with probe wash buffer at 37°C. Embryos were then washed with 5X saline sodium citrate containing 0.1% Tween-20 (5X SSCT) at room temperature, and then pre-amplified with amplification buffer (Molecular Instruments) for 30 min at room temperature. To prepare the hairpin mixture, 30 pmol of each hairpin (B3-Alexa Fluor^®^ 488, B1-Alexa-Fluor^®^ 546, B2-Alexa Fluor^®^ 647; Molecular Instruments) for each probe was snap cooled by incubation at 95°C for 90 s in a thermocycler then cooled to room temperature in the dark for 30 min. Snap-cooled hairpins were added to amplification buffer at room temperature to create the hairpin mixture. The pre-amplification solution was removed, embryos were immersed in the hairpin mixture, and then incubated for 12–16 h at room temperature protected from light. The next day, excess hairpins were removed by washing with 5X SSCT at room temperature. Samples were stored at 4°C in 1X PBS protected from light before microscopy. For qHCR, embryos were imaged within 24 h.

**Table 3.**
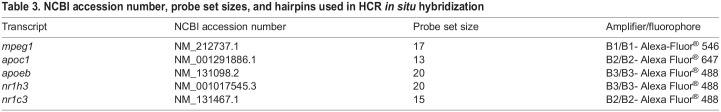
NCBI accession number, probe set sizes, and hairpins used in HCR *in situ* hybridization

### Microscopy and imaging

Images were acquired using a Nikon Andor spinning disk confocal microscope equipped with a Zyla sCMOS camera and computer running Nikon Elements software. Imaging was performed using a 20X air objective. Whole, fixed embryos were mounted in 0.7% agarose prepared in system water in glass bottom culture dishes (1.0 coverslip bottom, MatTek Corporation). Whole retinas were flattened and mounted on glass slides in Vectashield Vibrance (Vector Laboratories). For real-time imaging, embryos were dechorionated and anaesthetized with tricaine to prevent movement (0.01% w/v final concentration), then transferred to a glass bottom culture dish (1.0 coverslip bottom, MatTek Corporation). Excess water was removed, and the embryos were embedded in 1.5% agarose and oriented with ventral side on the bottom of the dish. Embryos in the solidified agarose were immersed in fresh water containing PTU and tricaine. During live imaging, dishes were placed in a temperature-controlled chamber (Okolab, set to 28°C). The top and bottom of the eye was identified using DIC optics, and time-lapse images were acquired using 5 µm z stacks (z, ∼100 µm total z depth) obtained every 5 min (t) for 8 h total. For whole fixed eyes and embryos, z stacks were obtained at 1–5 μm intervals, depending on the sample. Z stack images were visualized, with Nikon Elements software or ImageJ (Fiji). Movies were made using Nikon Elements software.

Imaging of embryos for qHCR was performed as described here, with all samples imaged on the same day and using identical acquisition settings (laser power, exposure, camera gain, and z stacks of 1 μm) for all samples. The embryos were first oriented for imaging of the optic tectum, then a soft paintbrush was used to re-orient them to image each eye. One control sample from the control group was used to determine optimal acquisition settings then excluded from downstream analysis.

### Quantification of HCR signal intensity in individual microglia (qHCR)

Images were imported into ImageJ (Fiji) for analysis. To prepare images, first color channels were split. The far-red channel, which most uniformly labeled the microglial cell body, was selected for tracing and duplicated. The duplicate image was imported into the segmentation editor and used to trace individual microglia. For each sample and tissue, 3–10 microglia were chosen for tracing. The perimeter of each selected cell was traced in individual z stack images and throughout the entire z-stack, being careful to choose cells that began and ended within the z-stack. Traced cells were saved as 3D objects in tiff format. Fluorescence intensity and geometrical measurements were then extracted for each object from each channel using the 3D plug-in in FIJI. Background signal intensity was measured in each channel by drawing regions of interest in locations where cells were unlabeled. Measurements were extracted to .csv file for further analysis using the R coding environment.

Quantitative *in situ* HCR (qHCR) analysis was modeled off the publication by Trivedi et al. (2018) with the modifications described here. All calculations were performed on a per cell and per channel basis. Total cell fluorescence (Integrated Density, IntDen) measurements were chosen for 3D analysis given that microglia are non-uniform in shape and size, and this signal should scale with total transcript levels on a per cell basis (Choi et al., 2018; Trivedi et al., 2018). To determine background signal for each traced cell in each channel, the mean fluorescence intensity of background was multiplied by the volume of the cell to determine the background signal per cell (BGcell). Corrected total cell fluorescence (TotalFluorCell) was then calculated by subtracting the background signal per cell from measured Integrated density (IntDenCell): TotalFluorCell=(IntDenCell)-(BGCell). An expression ratio compared to the control group was calculated for each transcript of interest by taking, for each fluorescent channel, the average TotalFluorCell of each cell divided by the mean TotalFluorCell from the cells of the control group, for retinas and optic tectum separately.

### Statistical Analysis

Statistical analysis and plotting were executed in the R coding environment. Levene's test and Shapiro-Wilk test were used to check ANOVA assumptions for homogeneity of variances and normality of data, respectively. Assumptions of normality and homogeneity were violated so non-parametric alternatives were used for analysis. For qPCR, qHCR data, and count data, Kruskal–Wallis rank sum test was used with post-hoc Dunn test to evaluate significant differences among treatments/groups. A *P*-value <0.05 was used as a cut off for significant differences, and *P*-values below this cut off are reported in the figures. Box plots of fold change by dose were created for each treatment using qPCR data for each transcript. Violin plots of normalized expression ratio compared to control by treatment were created to visualize HCR signal intensity analysis for each transcript of interest.

## Supplementary Material

Supplementary information

## References

[BIO058990C1] Abildayeva, K., Berbée, J. F. P., Blokland, A., Jansen, P. J., Hoek, F. J., Meijer, O., Lütjohann, D., Gautier, T., Pillot, T., Vente, J. D. et al. (2008). Human apolipoprotein C-I expression in mice impairs learning and memory functions. *J. Lipid Res.* 49, 856-869. 10.1194/jlr.M700518-JLR200 18160739

[BIO058990C2] A-Gonzalez, N., Bensinger, S. J., Hong, C., Beceiro, S., Bradley, M. N., Zelcer, N., Deniz, J., Ramirez, C., Díaz, M., Gallardo, G. et al. (2009). Apoptotic cells promote their own clearance and immune tolerance through activation of the nuclear receptor LXR. *Immunity* 31, 245-258. 10.1016/j.immuni.2009.06.018 19646905 PMC2791787

[BIO058990C3] Anderson, S. R., Zhang, J., Steele, M. R., Romero, C. O., Kautzman, A. G., Schafer, D. P. and Vetter, M. L. (2019). Complement targets newborn retinal ganglion cells for phagocytic elimination by microglia. *J. Neurosci. Official J. Soc. Neurosci.* 39, 2025-2040. 10.1523/JNEUROSCI.1854-18.2018 PMC6507095 30647151

[BIO058990C4] Archer, A., Lauter, G., Hauptmann, G., Mode, A. and Gustafsson, J. (2008). Transcriptional activity and developmental expression of liver X receptor (lxr) in Zebrafish. *Dev. Dynam* 237, 1090-1098. 10.1002/dvdy.21476 18297735

[BIO058990C5] Balducci, C., Paladini, A., Micotti, E., Tolomeo, D., Vitola, P. L., Grigoli, E., Richardson, J. C. and Forloni, G. (2015). The continuing failure of bexarotene in Alzheimer's disease mice. *J. Alzheimer's Dis.* 46, 471-482. 10.3233/JAD-150029 25777514

[BIO058990C6] Berbée, J. F. P., Vanmierlo, T., Abildayeva, K., Blokland, A., Jansen, P. J., Lütjohann, D., Gautier, T., Sijbrands, E., Prickaerts, J., Hadfoune, M. et al. (2011). Apolipoprotein CI knock-out mice display impaired memory functions. *J. Alzheimer's Dis.* 23, 737-747. 10.3233/JAD-2010-100576 21157034

[BIO058990C7] Bertram, L., McQueen, M. B., Mullin, K., Blacker, D. and Tanzi, R. E. (2007). Systematic meta-analyses of Alzheimer disease genetic association studies: the AlzGene database. *Nat. Genet.* 39, 17-23. 10.1038/ng1934 17192785

[BIO058990C8] Bilimoria, P. M. and Stevens, B. (2015). Microglia function during brain development: new insights from animal models. *Brain Res.* 1617, 7-17. 10.1016/j.brainres.2014.11.032 25463024

[BIO058990C9] Blume, Z. I., Lambert, J. M., Lovel, A. G. and Mitchell, D. M. (2020). Microglia in the developing retina couple phagocytosis with the progression of apoptosis via P2RY12 signaling. *Dev. Dynam.* 249, 723-740. 10.1002/dvdy.163 PMC8714022 32072708

[BIO058990C10] Brown, G. C. and Neher, J. J. (2014). Microglial phagocytosis of live neurons. *Nat. Rev. Neurosci.* 15, 209-216. 10.1038/nrn3710 24646669

[BIO058990C11] Casano, A. M., Albert, M. and Peri, F. (2016). Developmental apoptosis mediates entry and positioning of microglia in the Zebrafish brain. *Cell Rep.* 16, 897-906. 10.1016/j.celrep.2016.06.033 27425604

[BIO058990C12] Castellano, J. M., Kim, J., Stewart, F. R., Jiang, H., DeMattos, R. B., Patterson, B. W., Fagan, A. M., Morris, J. C., Mawuenyega, K. G., Cruchaga, C. et al. (2011). Human apoE isoforms differentially regulate brain amyloid-β peptide clearance. *Sci. Transl. Med.* 3, 89. 10.1126/scitranslmed.3002156 PMC3192364 21715678

[BIO058990C13] Cervantes, S., Samaranch, L., Vidal-Taboada, J. M., Lamet, I., Bullido, M. J., Frank-García, A., Coria, F., Lleó, A., Clarimón, J., Lorenzo, E. et al. (2011). Genetic variation in APOE cluster region and Alzheimer's disease risk. *Neurobiol. Aging* 32, 2107.e7-2107.e17. 10.1016/j.neurobiolaging.2011.05.023 21752496

[BIO058990C14] Chawla, A., Boisvert, W. A., Lee, C.-H., Laffitte, B. A., Barak, Y., Joseph, S. B., Liao, D., Nagy, L., Edwards, P. A., Curtiss, L. K. et al. (2001). A PPARγ-LXR-ABCA1 pathway in macrophages is involved in cholesterol efflux and atherogenesis. *Mol. Cell* 7, 161-171. 10.1016/S1097-2765(01)00164-2 11172721

[BIO058990C15] Chinetti, G., Lestavel, S., Bocher, V., Remaley, A. T., Neve, B., Torra, I. P., Teissier, E., Minnich, A., Jaye, M., Duverger, N. et al. (2001). PPAR-α and PPAR-γ activators induce cholesterol removal from human macrophage foam cells through stimulation of the ABCA1 pathway. *Nat. Med.* 7, 53-58. 10.1038/83348 11135616

[BIO058990C16] Choi, H. M. T., Schwarzkopf, M., Fornace, M. E., Acharya, A., Artavanis, G., Stegmaier, J., Cunha, A. and Pierce, N. A. (2018). Third-generation in situ hybridization chain reaction: multiplexed, quantitative, sensitive, versatile, robust. *Development* 145, dev165753. 10.1242/dev.165753 29945988 PMC6031405

[BIO058990C17] Collins, J. L., Fivush, A. M., Watson, M. A., Galardi, C. M., Lewis, M. C., Moore, L. B., Parks, D. J., Wilson, J. G., Tippin, T. K., Binz, J. G. et al. (2002). Identification of a nonsteroidal liver X receptor agonist through parallel array synthesis of tertiary amines. *J. Med. Chem.* 45, 1963-1966. 10.1021/jm0255116 11985463

[BIO058990C18] Cudaback, E., Li, X., Yang, Y., Yoo, T., Montine, K. S., Craft, S., Montine, T. J. and Keene, C. D. (2012). Apolipoprotein C-I is an APOE genotype-dependent suppressor of glial activation. *J. Neuroinflamm.* 9, 192. 10.1186/1742-2094-9-192 PMC3490924 22883744

[BIO058990C19] Cunningham, C. L., Martínez-Cerdeño, V. and Noctor, S. C. (2013). Microglia regulate the number of neural precursor cells in the developing cerebral cortex. *J. Neurosci.* 33, 4216-4233. 10.1523/JNEUROSCI.3441-12.2013 23467340 PMC3711552

[BIO058990C20] Deczkowska, A., Keren-Shaul, H., Weiner, A., Colonna, M., Schwartz, M. and Amit, I. (2018). Disease-associated microglia: a universal immune sensor of neurodegeneration. *Cell* 173, 1073-1081. 10.1016/j.cell.2018.05.003 29775591

[BIO058990C21] Diaz-Aparicio, I., Beccari, S., Abiega, O. and Sierra, A. (2016). Clearing the corpses: regulatory mechanisms, novel tools, and therapeutic potential of harnessing microglial phagocytosis in the diseased brain. *Neural. Regen. Res.* 11, 1533-1539. 10.4103/1673-5374.193220 27904472 PMC5116820

[BIO058990C22] Drigalenko, E., Poduslo, S. and Elston, R. (1998). Interaction of the apolipoprotein E and CI loci in predisposing to late-onset Alzheimer's disease. *Neurology* 51, 131-135. 10.1212/WNL.51.1.131 9674791

[BIO058990C23] Eastlake, K., Heywood, W. E., Tracey-White, D., Aquino, E., Bliss, E., Vasta, G. R., Mills, K., Khaw, P. T., Moosajee, M. and Limb, G. A. (2017). Comparison of proteomic profiles in the zebrafish retina during experimental degeneration and regeneration. *Sci. Rep.* 7, 44601. 10.1038/srep44601 28300160 PMC5353638

[BIO058990C24] Ebisawa, M., Umemiya, H., Ohta, K., Fukasawa, H., Kawachi, E., Christoffel, G., Gronemeyer, H., Tsuji, M., Hashimoto, Y., Shudo, K. et al. (1999). Retinoid X receptor-antagonistic diazepinylbenzoic acids. *Chem. Pharm. Bull.* 47, 1778-1786. 10.1248/cpb.47.1778 10748721

[BIO058990C25] Ellett, F., Pase, L., Hayman, J. W., Andrianopoulos, A. and Lieschke, G. J. (2011). mpeg1 promoter transgenes direct macrophage-lineage expression in zebrafish. *Blood* 117, e49-e56. 10.1182/blood-2010-10-314120 21084707 PMC3056479

[BIO058990C26] Evangelou, P., Groll, M., Oppermann, H., Gaunitz, F., Eisenlöffel, C., Müller, W., Eschrich, K., Schänzer, A. and Nestler, U. (2019). Assessment of ApoC1, LuzP6, C12orf75 and OCC-1 in cystic glioblastoma using MALDI–TOF mass spectrometry, immunohistochemistry and qRT-PCR. *Med. Mol. Morphol.* 52, 217-225. 10.1007/s00795-019-00223-8 31006040 PMC6885021

[BIO058990C27] Farré, D., Roset, R., Huerta, M., Adsuara, J. E., Roselló, L., Albà, M. M. and Messeguer, X. (2003). Identification of patterns in biological sequences at the ALGGEN server: PROMO and MALGEN. *Nucleic Acids Res.* 31, 3651-3653. 10.1093/nar/gkg605 12824386 PMC169011

[BIO058990C28] Dahabreh, D. F. and Medh, J. D. (2012). Activation of peroxisome proliferator activated receptor-gamma results in an atheroprotective apolipoprotein profile in HepG2 cells. *Adv. Biological. Chem.* 2012, 218-225. 10.4236/abc.2012.23026 PMC3632096 23616933

[BIO058990C29] Fitz, N. F., Nam, K. N., Koldamova, R. and Lefterov, I. (2019). Therapeutic targeting of nuclear receptors, liver X and retinoid X receptors, for Alzheimer's disease. *Brit. J. Pharmacol.* 176, 3599-3610. 10.1111/bph.14668 30924124 PMC6715597

[BIO058990C30] Fuior, E. V. and Gafencu, A. V. (2019). Apolipoprotein C1: its pleiotropic effects in lipid metabolism and beyond. *Int. J. Mol. Sci.* 20, 5939. 10.3390/ijms20235939 PMC6928722 31779116

[BIO058990C31] Galetto, R., Albajar, M., Polanco, J. I., Zakin, M. M. and Rodríguez-Rey, J. C. (2001). Identification of a peroxisome-proliferator-activated-receptor response element in the apolipoprotein E gene control region. *Biochem. J.* 357, 521. 10.1042/bj3570521 11439103 PMC1221980

[BIO058990C32] Germain, P., Chambon, P., Eichele, G., Evans, R. M., Lazar, M. A., Leid, M., Lera, A. R. D., Lotan, R., Mangelsdorf, D. J. and Gronemeyer, H. (2006). International union of pharmacology. LXIII. Retinoid X receptors. *Pharmacol. Rev.* 58, 760-772. 10.1124/pr.58.4.7 17132853

[BIO058990C33] Gosselin, D., Skola, D., Coufal, N. G., Holtman, I. R., Schlachetzki, J. C. M., Sajti, E., Jaeger, B. N., O'Connor, C., Fitzpatrick, C., Pasillas, M. P. et al. (2017). An environment-dependent transcriptional network specifies human microglia identity. *Science* 356, eaal3222. 10.1126/science.aal3222 28546318 PMC5858585

[BIO058990C34] Herbomel, P., Thisse, B. and Thisse, C. (2001). Zebrafish early macrophages colonize cephalic mesenchyme and developing brain, retina, and epidermis through a M-CSF receptor-dependent invasive process. *Dev. Biol.* 238, 274-288. 10.1006/dbio.2001.0393 11784010

[BIO058990C35] Herzog, C., Garcia, L. P., Keatinge, M., Greenald, D., Moritz, C., Peri, F. and Herrgen, L. (2019). Rapid clearance of cellular debris by microglia limits secondary neuronal cell death after brain injury in vivo. *Development* 146, dev174698. 10.1242/dev.174698 31076485 PMC6526721

[BIO058990C36] Holtman, I. R., Raj, D. D., Miller, J. A., Schaafsma, W., Yin, Z., Brouwer, N., Wes, P. D., Möller, T., Orre, M., Kamphuis, W. et al. (2015). Induction of a common microglia gene expression signature by aging and neurodegenerative conditions: a co-expression meta-analysis. *Acta Neuropathol. Commun.* 3, 31. 10.1186/s40478-015-0203-5 26001565 PMC4489356

[BIO058990C106] Hu, Y., Flockhart, I., Vinayagam, A., Bergwitz, C., Berger, B., Perrimon, N. and Mohr, S. E. (2011). An integrative approach to ortholog prediction for disease-focused and other functional studies. *BMC Bioinformatics* 12, 357. 21880147 10.1186/1471-2105-12-357 PMC3179972

[BIO058990C37] Joseph, S. B., McKilligin, E., Pei, L., Watson, M. A., Collins, A. R., Laffitte, B. A., Chen, M., Noh, G., Goodman, J., Hagger, G. N. et al. (2002). Synthetic LXR ligand inhibits the development of atherosclerosis in mice. *Proc. Natl. Acad. Sci.* 99, 7604-7609. 10.1073/pnas.112059299 12032330 PMC124297

[BIO058990C38] Jun, G., Vardarajan, B. N., Buros, J., Yu, C.-E., Hawk, M. V., Dombroski, B. A., Crane, P. K., Larson, E. B., Consortium, A. D. G., Mayeux, R. et al. (2012). Comprehensive search for Alzheimer disease susceptibility loci in the APOE region. *Arch. Neurol* 69, 1270-1279. 10.1001/archneurol.2012.2052 22869155 PMC3579659

[BIO058990C39] Kamijo, Y., Nicol, C. J. and Alexson, S. E. H. (2012). Pharmacological and toxicological advances in PPAR-related medicines. *Ppar. Res.* 2012, 940964. 10.1155/2012/940964 23125847 PMC3483781

[BIO058990C40] Kanayasu-Toyoda, T., Fujino, T., Oshizawa, T., Suzuki, T., Nishimaki-Mogami, T., Sato, Y., Sawada, J., Inoue, K., Shudo, K., Ohno, Y. et al. (2005). HX531, a retinoid X receptor antagonist, inhibited the 9-cis retinoic acid-induced binding with steroid receptor coactivator-1 as detected by surface plasmon resonance. *J. Steroid. Biochem. Mol. Biol.* 94, 303-309. 10.1016/j.jsbmb.2004.11.007 15857749

[BIO058990C41] Keren-Shaul, H., Spinrad, A., Weiner, A., Matcovitch-Natan, O., Dvir-Szternfeld, R., Ulland, T. K., David, E., Baruch, K., Lara-Astaiso, D., Toth, B. et al. (2017). A unique microglia type associated with restricting development of Alzheimer's disease. *Cell* 169, 1276-1290.e17. 10.1016/j.cell.2017.05.018 28602351

[BIO058990C42] Kim, J., Basak, J. M. and Holtzman, D. M. (2009). The role of apolipoprotein E in Alzheimer's disease. *Neuron* 63, 287-303. 10.1016/j.neuron.2009.06.026 19679070 PMC3044446

[BIO058990C43] Kiss, R. S., Elliott, M. R., Ma, Z., Marcel, Y. L. and Ravichandran, K. S. (2006). Apoptotic cells induce a phosphatidylserine-dependent homeostatic response from phagocytes. *Curr. Biol.* 16, 2252-2258. 10.1016/j.cub.2006.09.043 17113390

[BIO058990C44] Laffitte, B. A., Joseph, S. B., Walczak, R., Pei, L., Wilpitz, D. C., Collins, J. L. and Tontonoz, P. (2001a). Autoregulation of the human liver X receptor α promoter. *Mol. Cell. Biol.* 21, 7558-7568. 10.1128/MCB.21.22.7558-7568.2001 11604492 PMC99927

[BIO058990C45] Laffitte, B. A., Repa, J. J., Joseph, S. B., Wilpitz, D. C., Kast, H. R., Mangelsdorf, D. J. and Tontonoz, P. (2001b). LXRs control lipid-inducible expression of the apolipoprotein E gene in macrophages and adipocytes. *Proc. Natl. Acad. Sci. USA* 98, 507-512. 10.1073/pnas.98.2.507 11149950 PMC14617

[BIO058990C46] Lala, D. S., Mukherjee, R., Schulman, I. G., Koch, S. S. C., Dardashti, L. J., Nadzan, A. M., Croston, G. E., Evans, R. M. and Heyman, R. A. (1996). Activation of specific RXR heterodimers by an antagonist of RXR homodimers. *Nature* 383, 450-453. 10.1038/383450a0 8837780

[BIO058990C47] Lalloyer, F., Pedersen, T. Å., Gross, B., Lestavel, S., Yous, S., Vallez, E., Gustafsson, J.-Å., Mandrup, S., Fiévet, C., Staels, B. et al. (2009). Rexinoid bexarotene modulates triglyceride but not cholesterol metabolism via gene-specific permissivity of the RXR/LXR heterodimer in the liver. *Arterioscler. Thromb. Vasc Biol.* 29, 1488-1495. 10.1161/ATVBAHA.109.189506 19592467 PMC2824837

[BIO058990C48] Lauer, S. J., Walker, D., Elshourbagy, N. A., Reardon, C. A., Levy-Wilson, B. and Taylor, J. M. (1988). Two copies of the human apolipoprotein C-I gene are linked closely to the apolipoprotein E gene. *J. Biol. Chem.* 263, 7277-7286. 10.1016/S0021-9258(18)68638-7 2835369

[BIO058990C49] Liang, Y., Lin, S., Beyer, T. P., Zhang, Y., Wu, X., Bales, K. R., DeMattos, R. B., May, P. C., Li, S. D., Jiang, X. et al. (2004). A liver X receptor and retinoid X receptor heterodimer mediates apolipoprotein E expression, secretion and cholesterol homeostasis in astrocytes. *J. Neurochem.* 88, 623-634. 10.1111/j.1471-4159.2004.02183.x 14720212

[BIO058990C50] Mak, P. A., Laffitte, B. A., Desrumaux, C., Joseph, S. B., Curtiss, L. K., Mangelsdorf, D. J., Tontonoz, P. and Edwards, P. A. (2002). Regulated expression of the apolipoprotein E/C-I/C-IV/C-II gene cluster in murine and human macrophages a critical role for nuclear liver X receptors α and β*. *J. Biol. Chem.* 277, 31900-31908. 10.1074/jbc.M202993200 12032151

[BIO058990C51] Mathys, H., Davila-Velderrain, J., Peng, Z., Gao, F., Mohammadi, S., Young, J. Z., Menon, M., He, L., Abdurrob, F., Jiang, X. et al. (2019). Single-cell transcriptomic analysis of Alzheimer's disease. *Nature* 570, 332-337. 10.1038/s41586-019-1195-2 31042697 PMC6865822

[BIO058990C52] Mauch, D. H., Nägler, K., Schumacher, S., Göritz, C., Müller, E.-C., Otto, A. and Pfrieger, F. W. (2001). CNS synaptogenesis promoted by glia-derived cholesterol. *Science* 294, 1354-1357. 10.1126/science.294.5545.1354 11701931

[BIO058990C53] Mazaheri, F., Breus, O., Durdu, S., Haas, P., Wittbrodt, J., Gilmour, D. and Peri, F. (2014). Distinct roles for BAI1 and TIM-4 in the engulfment of dying neurons by microglia. *Nat. Commun.* 5, 4046. 10.1038/ncomms5046 24898390

[BIO058990C54] McCurley, A. T. and Callard, G. V. (2008). Characterization of housekeeping genes in zebrafish: male-female differences and effects of tissue type, developmental stage and chemical treatment. *BMC Mol. Biol.* 9, 102. 10.1186/1471-2199-9-102 19014500 PMC2588455

[BIO058990C55] Messeguer, X., Escudero, R., Farré, D., Núñez, O., Martínez, J. and Albà, M. M. (2002). PROMO: detection of known transcription regulatory elements using species-tailored searches. *Bioinformatics* 18, 333-334. 10.1093/bioinformatics/18.2.333 11847087

[BIO058990C56] Milinkeviciute, G., Henningfield, C. M., Muniak, M. A., Chokr, S. M., Green, K. N. and Cramer, K. S. (2019). Microglia regulate pruning of specialized synapses in the auditory brainstem. *Front. Neural Circuit* 13, 55. 10.3389/fncir.2019.00055 PMC6722190 31555101

[BIO058990C57] Mitchell, D. M., Stevens, C. B., Frey, R. A., Hunter, S. S., Ashino, R., Kawamura, S. and Stenkamp, D. L. (2015). Retinoic acid signaling regulates differential expression of the tandemly-duplicated long wavelength-sensitive cone opsin genes in Zebrafish. *PLoS Genet.* 11, e1005483. 10.1371/journal.pgen.1005483 26296154 PMC4546582

[BIO058990C58] Mitchell, D. M., Lovel, A. G. and Stenkamp, D. L. (2018). Dynamic changes in microglial and macrophage characteristics during degeneration and regeneration of the zebrafish retina. *J. Neuroinflamm.* 15, 163. 10.1186/s12974-018-1185-6 PMC5971432 29804544

[BIO058990C59] Mitchell, D. M., Sun, C., Hunter, S. S., New, D. D. and Stenkamp, D. L. (2019). Regeneration associated transcriptional signature of retinal microglia and macrophages. *Sci. Rep.* 9, 4768. 10.1038/s41598-019-41298-8 30886241 PMC6423051

[BIO058990C60] Neher, J. J., Neniskyte, U., Zhao, J.-W., Bal-Price, A., Tolkovsky, A. M. and Brown, G. C. (2011). Inhibition of microglial phagocytosis is sufficient to prevent inflammatory neuronal death. *J. Immunol.* 186, 4973-4983. 10.4049/jimmunol.1003600 21402900

[BIO058990C61] Neumann, H., Kotter, M. R. and Franklin, R. J. M. (2009). Debris clearance by microglia: an essential link between degeneration and regeneration. *Brain* 132, 288-295. 10.1093/brain/awn109 18567623 PMC2640215

[BIO058990C107] Nieto-Arellano, R. and Sánchez-Iranzo, H. (2018). zfRegeneration: a database for gene expression profiling during regeneration. *Bioinformatics* 35, 703-705. 10.1093/bioinformatics/bty659 30052798

[BIO058990C62] Ogata, M., Tsujita, M., Hossain, M. A., Akita, N., Gonzalez, F. J., Staels, B., Suzuki, S., Fukutomi, T., Kimura, G. and Yokoyama, S. (2009). On the mechanism for PPAR agonists to enhance ABCA1 gene expression. *Atherosclerosis* 205, 413-419. 10.1016/j.atherosclerosis.2009.01.008 19201410 PMC2790138

[BIO058990C63] Olah, M., Patrick, E., Villani, A.-C., Xu, J., White, C. C., Ryan, K. J., Piehowski, P., Kapasi, A., Nejad, P., Cimpean, M. et al. (2018). A transcriptomic atlas of aged human microglia. *Nat. Commun.* 9, 539. 10.1038/s41467-018-02926-5 29416036 PMC5803269

[BIO058990C64] Oosterhof, N., Holtman, I. R., Kuil, L. E., van der Linde, H. C., Boddeke, E. W. G. M., Eggen, B. J. L. and van Ham, T. J. (2017). Identification of a conserved and acute neurodegeneration–specific microglial transcriptome in the zebrafish. *Glia* 65, 138-149. 10.1002/glia.23083 27757989 PMC5215681

[BIO058990C65] Otis, J. P., Zeituni, E. M., Thierer, J. H., Anderson, J. L., Brown, A. C., Boehm, E. D., Cerchione, D. M., Ceasrine, A. M., Avraham-Davidi, I., Tempelhof, H. et al. (2015). Zebrafish as a model for apolipoprotein biology: comprehensive expression analysis and a role for ApoA-IV in regulating food intake. *Dis. Model. Mech.* 8, 295-309. 10.1242/dmm.018754 25633982 PMC4348566

[BIO058990C66] Paolicelli, R. C., Bolasco, G., Pagani, F., Maggi, L., Scianni, M., Panzanelli, P., Giustetto, M., Ferreira, T. A., Guiducci, E., Dumas, L. et al. (2011). Synaptic pruning by microglia is necessary for normal brain development. *Science* 333, 1456-1458. 10.1126/science.1202529 21778362

[BIO058990C67] Peri, F. and Nüsslein-Volhard, C. (2008). Live imaging of neuronal degradation by microglia reveals a role for v0-ATPase a1 in phagosomal fusion In Vivo. *Cell* 133, 916-927. 10.1016/j.cell.2008.04.037 18510934

[BIO058990C68] Perz-Edwards, A., Hardison, N. L. and Linney, E. (2001). Retinoic acid-mediated gene expression in transgenic reporter Zebrafish. *Dev. Biol.* 229, 89-101. 10.1006/dbio.2000.9979 11133156

[BIO058990C69] Petit-Turcotte, C., Stohl, S. M., Beffert, U., Cohn, J. S., Aumont, N., Tremblay, M., Dea, D., Yang, L., Poirier, J. and Shachter, N. S. (2001). Apolipoprotein C-I expression in the brain in Alzheimer's disease. *Neurobiol. Dis.* 8, 953-963. 10.1006/nbdi.2001.0441 11741391

[BIO058990C70] Pinto, C. L., Kalasekar, S. M., McCollum, C. W., Riu, A., Jonsson, P., Lopez, J., Swindell, E. C., Bouhlatouf, A., Balaguer, P., Bondesson, M. et al. (2016). Lxr regulates lipid metabolic and visual perception pathways during zebrafish development. *Mol. Cell. Endocrinol.* 419, 29-43. 10.1016/j.mce.2015.09.030 26427652 PMC4684448

[BIO058990C71] Poduslo, S. E., Neal, M., Herring, K. and Shelly, J. (1998). The apolipoprotein CI A Allele as a risk factor for Alzheimer's disease. *Neurochem. Res.* 23, 361-367. 10.1023/A:1022409617539 9482248

[BIO058990C72] Prabhudesai, S. N., Cameron, D. A. and Stenkamp, D. L. (2005). Targeted effects of retinoic acid signaling upon photoreceptor development in zebrafish. *Dev. Biol.* 287, 157-167. 10.1016/j.ydbio.2005.08.045 16197938 PMC2804901

[BIO058990C73] Prendecki, M., Florczak-Wyspianska, J., Kowalska, M., Ilkowski, J., Grzelak, T., Bialas, K., Wiszniewska, M., Kozubski, W. and Dorszewska, J. (2018). Biothiols and oxidative stress markers and polymorphisms of TOMM40 and APOC1 genes in Alzheimer's disease patients. *Oncotarget* 9, 35207-35225. 10.18632/oncotarget.26184 30443289 PMC6219666

[BIO058990C74] Raymond, P. A., Barthel, L. K., Bernardos, R. L. and Perkowski, J. J. (2006). Molecular characterization of retinal stem cells and their niches in adult zebrafish. *BMC Dev. Biol.* 6, 36. 10.1186/1471-213X-6-36 16872490 PMC1564002

[BIO058990C75] Repa, J. J., Turley, S. D., Lobaccaro, J.-M. A., Medina, J., Li, L., Lustig, K., Shan, B., Heyman, R. A., Dietschy, J. M. and Mangelsdorf, D. J. (2000). Regulation of absorption and ABC1-mediated efflux of cholesterol by RXR heterodimers. *Science* 289, 1524-1529. 10.1126/science.289.5484.1524 10968783

[BIO058990C76] Reschly, E. J., Ai, N., Welsh, W. J., Ekins, S., Hagey, L. R. and Krasowski, M. D. (2008). Ligand specificity and evolution of liver X receptors. *J. Steroid. Biochem. Mol. Biol.* 110, 83-94. 10.1016/j.jsbmb.2008.02.007 18395439 PMC2519238

[BIO058990C77] Saunders, A., Hulette, C., Welsh-Bohmer, K., Schmechel, D., Crain, B., Burke, J., Alberts, M., Strittmatter, W., Breitner, J., Rosenberg, C. et al. (1996). Specificity, sensitivity, and predictive value of apolipoprotein-E genotyping for sporadic Alzheimer's disease. *Lancet* 348, 90-93. 10.1016/S0140-6736(96)01251-2 8676723

[BIO058990C78] Schaaf, M. J. M. (2017). Nuclear receptor research in zebrafish. *J. Mol. Endocrinol.* 59, R65-R76. 10.1530/JME-17-0031 28438785

[BIO058990C79] Schafer, D. P., Lehrman, E. K., Kautzman, A. G., Koyama, R., Mardinly, A. R., Yamasaki, R., Ransohoff, R. M., Greenberg, M. E., Barres, B. A. and Stevens, B. (2012). Microglia sculpt postnatal neural circuits in an activity and complement-dependent manner. *Neuron* 74, 691-705. 10.1016/j.neuron.2012.03.026 22632727 PMC3528177

[BIO058990C80] Schulman, I. G., Li, C., Schwabe, J. W. and Evans, R. M. (1997). The phantom ligand effect: allosteric control of transcription by the retinoid X receptor. *Gene Dev* 11, 299-308. 10.1101/gad.11.3.299 9030683

[BIO058990C81] Scott-Hewitt, N., Perrucci, F., Morini, R., Erreni, M., Mahoney, M., Witkowska, A., Carey, A., Faggiani, E., Schuetz, L. T., Mason, S. et al. (2020). Local externalization of phosphatidylserine mediates developmental synaptic pruning by microglia. *EMBO J.* 39, e105380. 10.15252/embj.2020105380 32657463 PMC7429741

[BIO058990C82] Sieger, D., Moritz, C., Ziegenhals, T., Prykhozhij, S. and Peri, F. (2012). Long-Range Ca2+ waves transmit brain-damage signals to microglia. *Dev. Cell* 22, 1138-1148. 10.1016/j.devcel.2012.04.012 22632801

[BIO058990C83] Sierra, A., Encinas, J. M., Deudero, J. J. P., Chancey, J. H., Enikolopov, G., Overstreet-Wadiche, L. S., Tsirka, S. E. and Maletic-Savatic, M. (2010). Microglia shape adult hippocampal neurogenesis through apoptosis-coupled phagocytosis. *Cell Stem. Cell* 7, 483-495. 10.1016/j.stem.2010.08.014 20887954 PMC4008496

[BIO058990C84] Smit, M., van der Kooij-Meijs, E., Frants, R. R., Havekes, L. and Klasen, E. C. (1988). Apolipoprotein gene cluster on chromosome 19. *Hum. Genet.* 78, 90-93. 10.1007/BF00291243 2892779

[BIO058990C108] Stevens, C. B., Cameron, D. A. and Stenkamp, D. L. (2011). Plasticity of photoreceptor-generating retinal progenitors revealed by prolonged retinoic acid exposure. *BMC Dev. Biol.* 11, 51-51. 21878117 10.1186/1471-213X-11-51 PMC3189157

[BIO058990C85] Srinivasan, K., Friedman, B. A., Etxeberria, A., Huntley, M. A., van der Brug, M. P., Foreman, O., Paw, J. S., Modrusan, Z., Beach, T. G., Serrano, G. E. et al. (2020). Alzheimer's patient microglia exhibit enhanced aging and unique transcriptional activation. *Cell Reports* 31, 107843. 10.1016/j.celrep.2020.107843 32610143 PMC7422733

[BIO058990C86] Strittmatter, W. J., Saunders, A. M., Schmechel, D., Pericak-Vance, M., Enghild, J., Salvesen, G. S. and Roses, A. D. (1993). Apolipoprotein E: high-avidity binding to beta-amyloid and increased frequency of type 4 allele in late-onset familial Alzheimer disease. *Proc. Natl. Acad. Sci. USA* 90, 1977-1981. 10.1073/pnas.90.5.1977 8446617 PMC46003

[BIO058990C87] Subramanian, S., Gottschalk, W. K., Kim, S. Y., Roses, A. D. and Chiba-Falek, O. (2017). The effects of PPARγ on the regulation of the TOMM40-APOE-C1 genes cluster. *Biochim. Biophys. Acta Mol. Basis Dis.* 1863, 810-816. 10.1016/j.bbadis.2017.01.004 28065845 PMC5285471

[BIO058990C88] Tousi, B. (2015). The emerging role of bexarotene in the treatment of Alzheimer's disease: current evidence. *Neuropsychiatr. Dis. Treat* 11, 311-315. 10.2147/NDT.S61309 25709453 PMC4327563

[BIO058990C89] Tracey, T. J., Steyn, F. J., Wolvetang, E. J. and Ngo, S. T. (2018). Neuronal lipid metabolism: multiple pathways driving functional outcomes in health and disease. *Front. Mol. Neurosci.* 11, 10. 10.3389/fnmol.2018.00010 29410613 PMC5787076

[BIO058990C90] Trivedi, V., Choi, H. M. T., Fraser, S. E. and Pierce, N. A. (2018). Multidimensional quantitative analysis of mRNA expression within intact vertebrate embryos. *Development* 145, dev156869. 10.1242/dev.156869 29311262 PMC5825878

[BIO058990C91] Verghese, P. B., Castellano, J. M. and Holtzman, D. M. (2011). Apolipoprotein E in Alzheimer's disease and other neurological disorders. *Lancet Neurol.* 10, 241-252. 10.1016/S1474-4422(10)70325-2 21349439 PMC3132088

[BIO058990C92] Verghese, P. B., Castellano, J. M., Garai, K., Wang, Y., Jiang, H., Shah, A., Bu, G., Frieden, C. and Holtzman, D. M. (2013). ApoE influences amyloid-β (Aβ) clearance despite minimal apoE/Aβ association in physiological conditions. *Proc. Natl. Acad. Sci. USA* 110, E1807-E1816. 10.1073/pnas.1220484110 23620513 PMC3651443

[BIO058990C93] Vilalta, A. and Brown, G. C. (2018). Neurophagy, the phagocytosis of live neurons and synapses by glia, contributes to brain development and disease. *FEBS J.* 285, 3566-3575. 10.1111/febs.14323 29125686

[BIO058990C94] Vivat, V., Zechel, C., Wurtz, J., Bourguet, W., Kagechika, H., Umemiya, H., Shudo, K., Moras, D., Gronemeyer, H. and Chambon, P. (1997). A mutation mimicking ligand–induced conformational change yields a constitutive RXR that senses allosteric effects in heterodimers. *EMBO J.* 16, 5697-5709. 10.1093/emboj/16.18.5697 9312028 PMC1170201

[BIO058990C95] Wang, H. and Eckel, R. H. (2014). What are lipoproteins doing in the brain? *Trends Endocrinol Metab.* 25, 8-14. 10.1016/j.tem.2013.10.003 24189266 PMC4062975

[BIO058990C96] Wang, Y., Zhou, L., Li, Z., Li, W. and Gui, J. (2013). Apolipoprotein C1 regulates epiboly during gastrulation in zebrafish. *Sci. China Life Sci.* 56, 975-984. 10.1007/s11427-013-4563-4 24203452

[BIO058990C97] Wang, Y., Li, W.-H., Li, Z., Liu, W., Zhou, L. and Gui, J.-F. (2015). BMP and RA signaling cooperate to regulate Apolipoprotein C1 expression during embryonic development. *Gene* 554, 196-204. 10.1016/j.gene.2014.10.047 25445289

[BIO058990C98] Weinhard, L., di Bartolomei, G., Bolasco, G., Machado, P., Schieber, N. L., Neniskyte, U., Exiga, M., Vadisiute, A., Raggioli, A., Schertel, A. et al. (2018). Microglia remodel synapses by presynaptic trogocytosis and spine head filopodia induction. *Nat. Commun.* 9, 1228. 10.1038/s41467-018-03566-5 29581545 PMC5964317

[BIO058990C110] Westerfield, M. (2007). *The Zebrafish Book. A Guide for the Laboratory Use of Zebrafish (Danio rerio), 5th Edition*. Eugene: University of Oregon Press.

[BIO058990C99] Wu, S., Nguyen, L. T. M., Pan, H., Hassan, S., Dai, Y., Xu, J. and Wen, Z. (2020). Two phenotypically and functionally distinct microglial populations in adult zebrafish. *Sci. Adv.* 6, eabd1160. 10.1126/sciadv.abd1160 33208372 PMC7673811

[BIO058990C100] Xu, J., Wang, T., Wu, Y., Jin, W. and Wen, Z. (2016). Microglia colonization of developing Zebrafish midbrain is promoted by apoptotic neuron and Lysophosphatidylcholine. *Dev. Cell* 38, 214-222. 10.1016/j.devcel.2016.06.018 27424497

[BIO058990C101] Yin, J., Spillman, E., Cheng, E. S., Short, J., Chen, Y., Lei, J., Gibbs, M., Rosenthal, J. S., Sheng, C., Chen, Y. X. et al. (2021). Brain-specific lipoprotein receptors interact with astrocyte derived apolipoprotein and mediate neuron-glia lipid shuttling. *Nat. Commun.* 12, 2408. 10.1038/s41467-021-22751-7 33893307 PMC8065144

[BIO058990C102] Zelcer, N., Khanlou, N., Clare, R., Jiang, Q., Reed-Geaghan, E. G., Landreth, G. E., Vinters, H. V. and Tontonoz, P. (2007). Attenuation of neuroinflammation and Alzheimer's disease pathology by liver x receptors. *Proc. Natl. Acad. Sci. USA* 104, 10601-10606. 10.1073/pnas.0701096104 17563384 PMC1890560

[BIO058990C103] Zhao, J., Fu, Y., Liu, C.-C., Shinohara, M., Nielsen, H. M., Dong, Q., Kanekiyo, T. and Bu, G. (2014). Retinoic acid isomers facilitate apolipoprotein E production and lipidation in astrocytes through the retinoid X receptor/retinoic acid receptor pathway*. *J. Biol. Chem.* 289, 11282-11292. 10.1074/jbc.M113.526095 24599963 PMC4036266

[BIO058990C104] Zhou, Q., Zhao, F., Lv, Z., Zheng, C., Zheng, W., Sun, L., Wang, N., Pang, S., de Andrade, F. M., Fu, M. et al. (2014). Association between APOC1 polymorphism and Alzheimer's disease: a case-control study and meta-analysis. *PLoS ONE* 9, e87017. 10.1371/journal.pone.0087017 24498013 PMC3909044

[BIO058990C105] Zhou, X., Chen, Y., Mok, K. Y., Kwok, T. C. Y., Mok, V. C. T., Guo, Q., Ip, F. C., Chen, Y., Mullapudi, N., Weiner, M. W. et al. (2019). Non-coding variability at the APOE locus contributes to the Alzheimer's risk. *Nat. Commun.* 10, 3310. 10.1038/s41467-019-10945-z 31346172 PMC6658518

