# Supplementary material for: Modulation of retinoid-X-receptors differentially regulates expression of apolipoprotein genes apoc1 and apoeb by zebrafish microglia
Source: Biol Open. 2022 Feb 1;11(1):bio058990. doi: 10.1242/bio.058990 (PMC8822359; doi:10.1242/bio.058990)
Supplement: Supplementary information [file biolopen-11-058990-s1.pdf]

Tool	Simple Score	Weighted Score	Original Tool Information			
			BLAST Result	Score	Score Type	Cluster ID
Compara	1	0.930				C1151282
eggNOG	1	0.900				E1_2E8B8
Hieranoid	1	1.000				
Homologene	0	0.000	*Not matched by this tool.*			
Inparanoid	1	1.050	53	1.000	Inparanoid score	I12312
OMA	0	0.000	*Not matched by this tool.*			
OrthoDB	0	0.000	*Not matched by this tool.*			
OrthoFinder	1	1.000				FOG0006587
OrthoInspector	1	1.000				T1603566
orthoMCL	0	0.000	*Not matched by this tool.*			
Panther	1	1.100			LDO	PTHR16565
Phylome	0	0.000	*Not matched by this tool.*			
RoundUp	1	1.030		avgDist	Average_Evolutionary_Distance	R8175
TreeFam	1	0.960				
ZFIN	1	1.500				
	**10**	**10.470**				

Input Order	Search Term	Zebrafish GeneID	ZFINID	Zebrafish Symbol	Species 2	Human GeneID	Human Species Gene ID	Human Symbol	DIOPT Score	Weighted Score	Rank	Best Score	Best Score Reverse	Prediction Derived From	Alignment & Scores	Feedback	Gene2Function Details
1	apoc1	570638	ZDB-GENE-030131-1074	apoc1	Human	341	607	APOC1	10	10.47	high	Yes	Yes	Compara, eggNOG, Hieranoid, Inparanoid, OrthoFinder, OrthoInspector, Panther, RoundUp, TreeFam, ZFIN	View	Add	APOC1 details

Fig. S1. Output data from the DRSC Integrative Ortholog Prediction Tool. The different ortholog prediction algorithms that DIOPT compares are shown in the left column. The simple score from each algorithm predicts whether *apoc1* is orthologous (score=1) or not (score=0) to human *APOC1*. The weighted score is based on each of the algorithms prediction score that DIOPT assigns. Bottom, summary of result with ranking and scoring.

Biology Open • Supplementary information

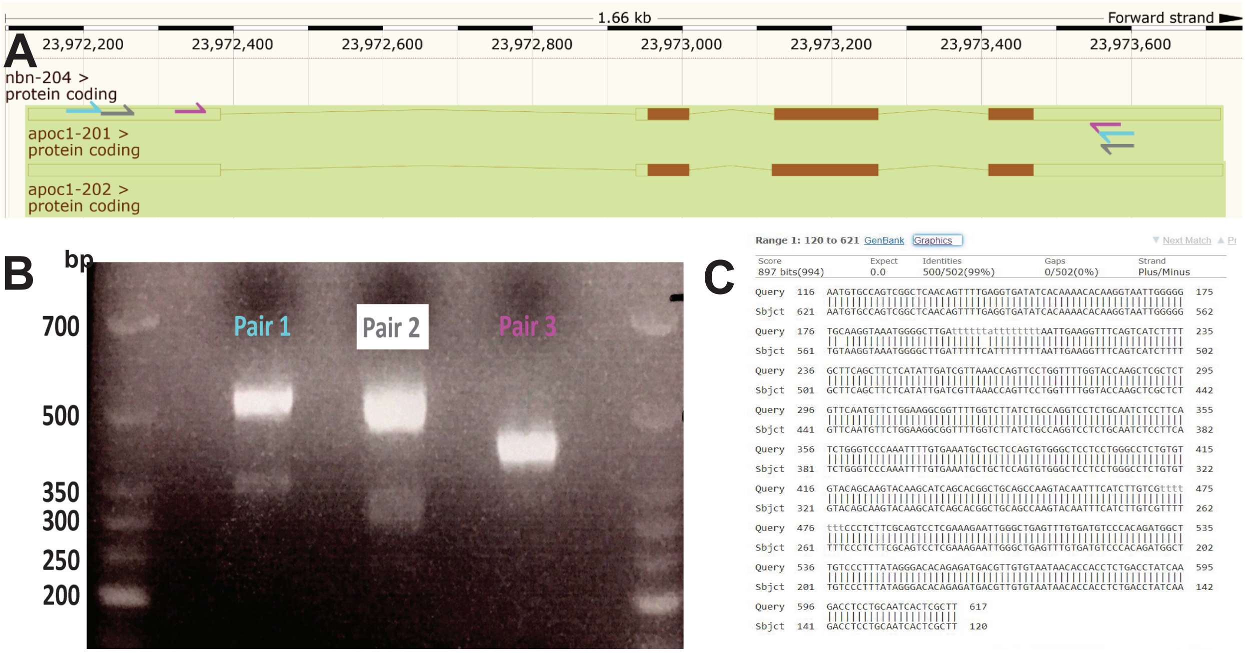

Fig. S2. A. PCR primers (3 pairs, indicated by different colors) used to synthesize *apoc1* cDNA from retinal mRNA after reverse transcription and the approximate location of annealing on the *apoc1* coding sequence. B. Gel electrophoresis after RT-PCR returning amplicons at expected sizes for *apoc1* cDNA. C. Sanger sequencing results of amplicon obtained from Primer Pair 2 and alignment to *apoc1* coding sequence.

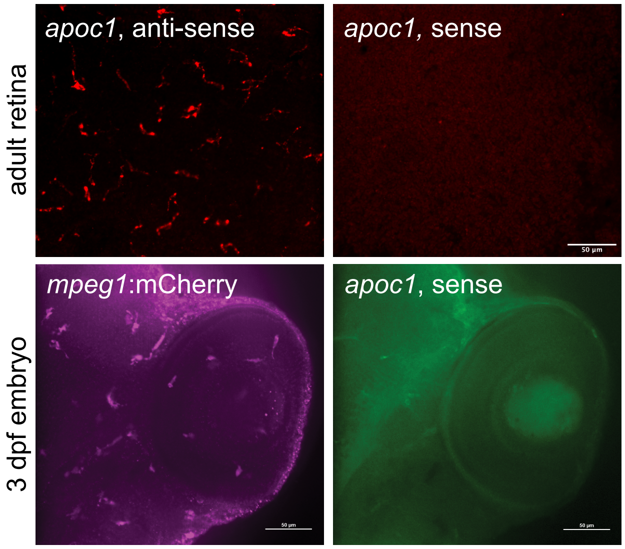

Fig. S3. Validation of in-house generated RNA probes to detect *apoc1* transcripts in situ. Images from adult retina (top row) and head of 3 dpf *mpeg1*:mCherry transgenic embryo (bottom row). Signal is only obtained with the anti-sense probe.

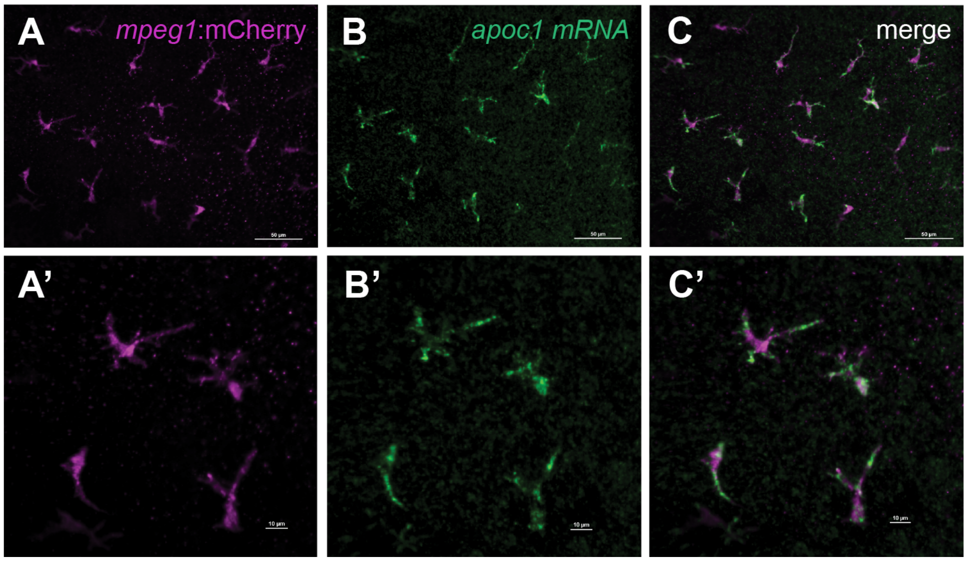

Fig. S4. Images from regions of flat-mounted whole adult zebrafish retinas showing expression of mpeg1:mCherry reporter (A, magenta) in microglia and *apoc1* transcripts detected by in-house generated RNA probes (B, green). Merged images shown in C. A', B', and C' show enlarged views of A-C.

Biology Open • Supplementary information

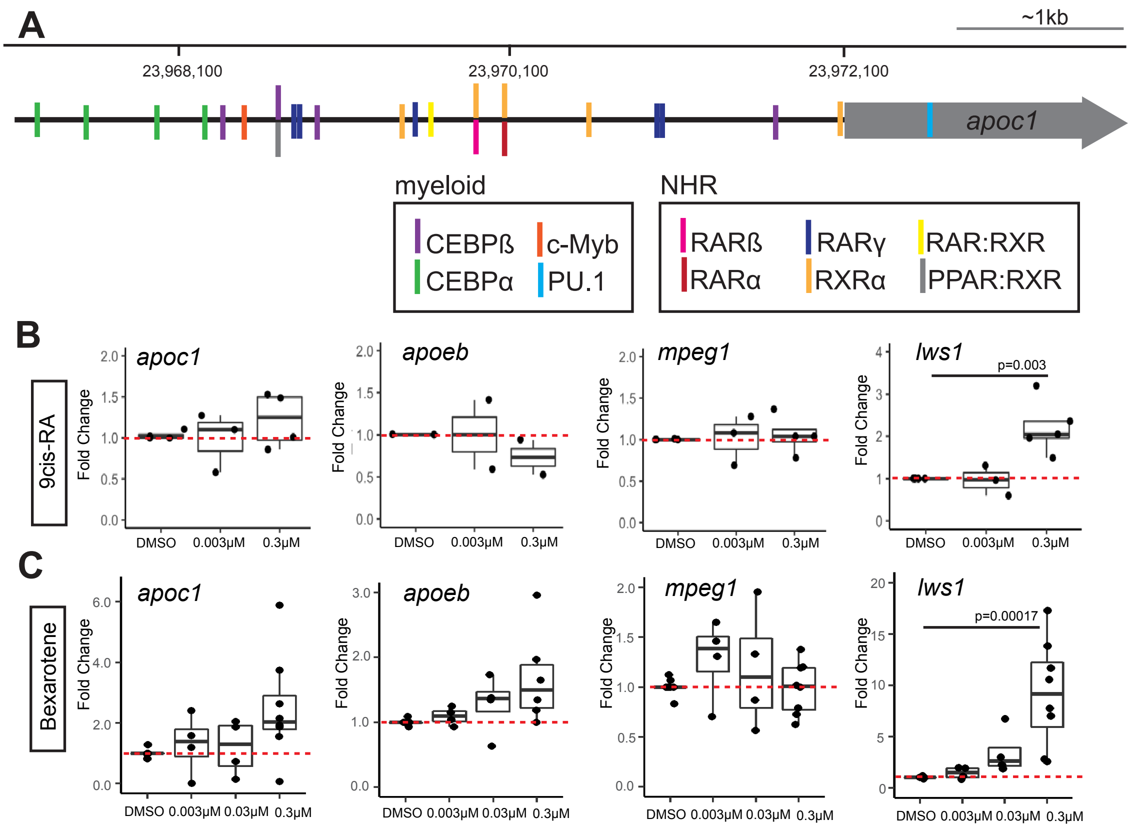

Fig. S5. A. Results of PROMO analysis showing returned predicted transcription factor binding sites in the zebrafish *apoc1* upstream genomic region. B. Effects of 9-cisRA on the indicated genes measured by qPCR, using RNA isolated from whole embryo heads. C. Effects of Bexarotene on the indicated genes measured by qPCR, using RNA isolated from whole embryo heads. Each dot represents the value from one sample of pooled heads (3-9 heads pooled per sample). Statistically significant differences (only found for the control gene, *lws1*) are indicated by shown p-values.

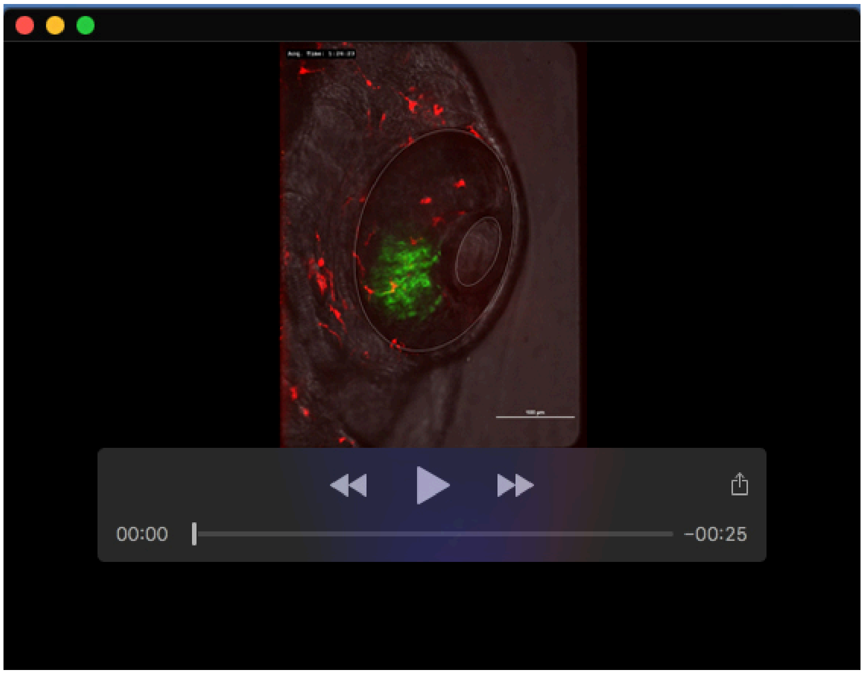

Movie 1. Real-time live imaging of brain and eye of *RARE*:YFP; *mpeg1*:mCherry double transgenic embryo at 3 dpf. Microglia (mCherry+) migrate through the active RA signaling zone (YFP+, green) but do not express the *RARE*:YFP reporter. Outlines mark the eye and lens. Imaging over a total of 8 hours, with z stacks acquired every 5 minutes. Timestamp in upper left is in hours:min:sec.
